# River Blindness: A Success Story under Threat?

**DOI:** 10.1371/journal.pmed.0030371

**Published:** 2006-09-26

**Authors:** María-Gloria Basáñez, Sébastien D. S Pion, Thomas S Churcher, Lutz P Breitling, Mark P Little, Michel Boussinesq

## Abstract

The success of the Onchocerciasis Control Programme is undeniable and exemplary, say the authors, but it is too early to claim victory against river blindness.

“The accomplishments of this Programme inspire all of us in public health to dream big dreams. It shows we can reach ‘impossible’ goals and lighten the burden of millions of the world's poorest people ….” These were the concluding words by former World Health Organization Director-General Gro Harlem Brundtland at the closure ceremony of the Onchocerciasis Control Programme in West Africa (OCP) in December 2002 [1]. The success of the OCP is so undeniable and exemplary, with 600,000 cases of blindness prevented, 18 million children born in areas freed from the risk of blindness, and 25 million hectares of land safe for resettlement, that river blindness is currently considered a disease of the past. This perception nonetheless forgets that OCP covered, at most, 1,200,000 square kilometers to protect 30 million people in 11 countries, leaving a remaining 100 million people in areas where active transmission of onchocerciasis still occurs. After its 28-year fight OCP may have won a battle, but a much more difficult task lies ahead before we can claim victory against river blindness [2].

## Etiology and Distribution

**Figure 1:**
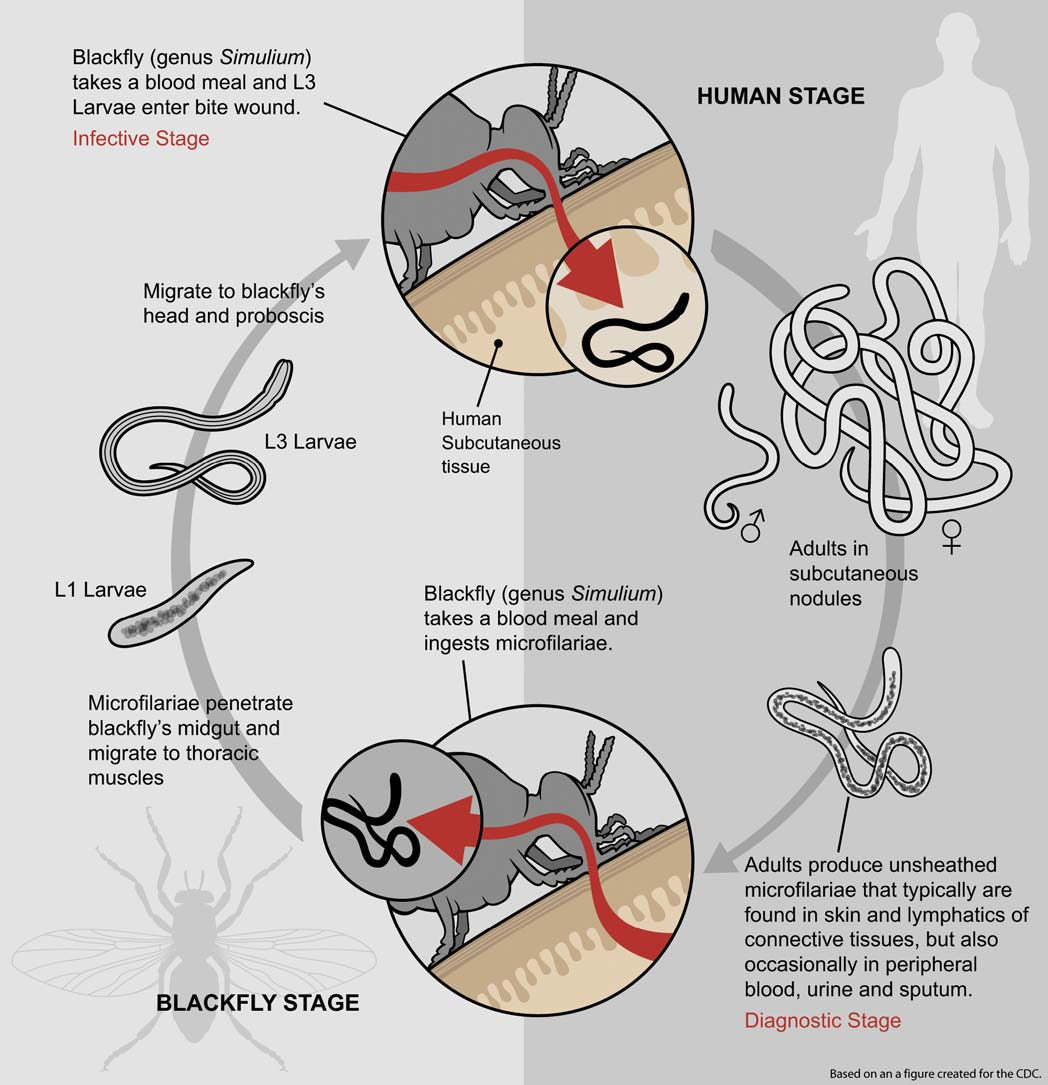
Life Cycle of *O. volvulus* Mean dimensions of parasite stages are: Adult females, 35–70 cm × 400 µm; adult males, 2–4 cm × 150–200 µm; microfilariae, 250–360 × 5–9 µm; L1 larvae, 200 µm × 12 µm (front) and 20 µm (rear); L3, 440–700 × 20 µm. L1 larvae molt into L2, pre-infective larvae, and L2 into L3, infective larvae [5]. (Illustration: Giovanni Maki, derived from a CDC image at http://www.dpd.cdc.gov/dpdx/HTML/Filariasis.htm)

Human onchocerciasis is caused by the filarial parasitic nematode *Onchocerca volvulus*. Adult worms (macrofilariae) live in subcutaneous nodules and deeper worm bundles, where fertilized females can produce, during an average of 10 years, millions of microfilariae responsible for the morbidity associated with the infection. Ingested during a bloodmeal by *Simulium* (black fly) vectors, microfilariae develop within the fly to infective (L3) stages, that are then transmissible to other people (Figure 1). Many simuliid species have been incriminated to a greater or lesser degree in the transmission of *O. volvulus* [3], their relative vectorial roles contributing to shape diverse transmission patterns across endemic areas. In Africa, the *Simulium damnosum* sensu lato (s.l.) species complex, which includes approximately 60 cytoforms, is responsible for more than 95 percent of onchocerciasis cases globally [3,4]. In Latin America, *S. ochraceum*s.l., *S. exiguum*s.l., *S. metallicum*s.l., and *S. guianense*s.l. are the main vectors, respectively, in Mexico and Guatemala (about 360,000 people at risk), Colombia and Ecuador (24,600), northern Venezuela (104,500), and southern Venezuela and Brazil (20,000) [5,6]

**Figure 2:**
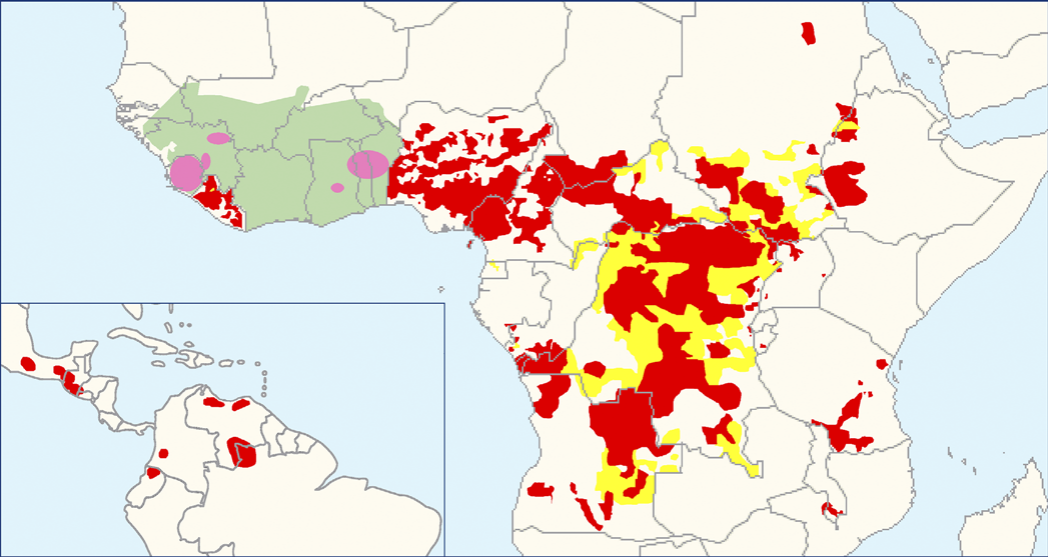
Distribution of Onchocerciasis Showing Current Status of Global Onchocerciasis Control Red areas represent areas receiving ivermectin treatment. Yellow areas represent areas requiring further epidemiological surveys. The green area is the area covered by the Onchocerciasis Control Programme in West Africa. Pink zones indicate the special intervention zones, i.e., previous OCP areas receiving ivermectin and some vector control. Map redrawn from [53,75,76]

*O. volvulus*is endemic in 27 sub-Saharan African countries, the Yemen [7], and was imported through the slave trade to six Latin American countries. Previous estimates have placed the number of people infected worldwide at 18 million [7], 99 percent of them in Africa. Since then, the true extent of the disease has been estimated by REMO (rapid epidemiological mapping of onchocerciasis). Villages are selected in each river basin according to appropriate criteria, and levels of endemicity are assessed by onchocercal nodule prevalence in adult host samples [8]. By 2005, more than 22,000 villages in Africa (outside the OCP area) had been surveyed, allowing the identification of many new foci (Figure 2). The new infected populations thus found, together with their demographic increase, certainly compensate for the number of cases prevented by the OCP (where it was estimated that roughly 3 million people were infected [7]). Presently, it is estimated that 37 million people carry *O. volvulus*, with 90 million at risk in Africa [9].

## Clinical Manifestations and Pathogenesis

**Figure 3:**
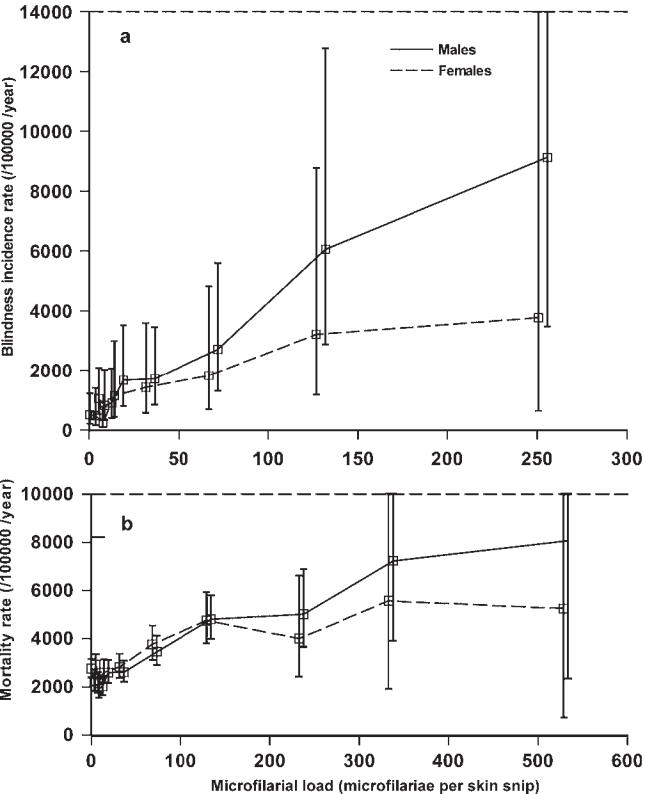
The Incidence of Blindness and Excess Mortality Rate, by Sex, Plotted against *O. volvulus* Microfilarial Load Arithmetic mean of microfilarial counts from two skin snips, taken from the right and left ileac crests, using a 2-millimeter Holth corneoscleral punch. (A) Blindness; (B) excess mortality rate. Error bars denote 95 percent confidence intervals [10,22].

Onchocerciasis is better known as river blindness because of the high prevalence of blindness in villages located along fast-flowing rivers, where the vectors breed. Up to 500,000 cases of severe visual impairment (including visual field reduction), and 270,000 of blindness have been attributed to onchocerciasis [7], but, again, these figures certainly underestimate the true magnitude of the problem. Ocular lesions can involve all eye tissues, ranging from punctate and sclerosing keratitis (anterior segment) to optic nerve atrophy (posterior segment). Blindness incidence has recently been shown to be associated with past microfilarial load in individuals followed up within the OCP cohort [10], confirming the progressive worsening of onchocercal eye disease with parasite exposure (Figure 3A). Conventionally, anterior chamber lesions had been attributed to a cascade of inflammatory processes triggered by filarial products [11]. A novel hypothesis proposes that the proinflammatory events leading to increasing corneal opacity are stimulated not only by the parasite itself, but also by its recently discovered endosymbiotic *Wolbachia*bacteria, when released by dying microfilariae [12,13]. By contrast, the pathogenesis of retinal lesions, which may continue progressing despite parasite clearance after chemotherapy, may result from autoimmune processes elicited by cross-reactivity between the *O. volvulus*antigen Ov39 and the human retinal antigen hr44 [14].

Onchocerciasis also causes troublesome itching and skin changes ranging from early, reactive lesions—acute papular onchodermatitis, chronic papular onchodermatitis, and lichenified onchodermatitis—to late changes such as depigmentation and skin atrophy [15]. When limited to one limb, lichenified onchodermatitis is also called “sowda.” Despite high skin microfilarial loads in endemic areas, most patients present with subclinical or intermittent dermatitis corresponding to acute papular onchodermatitis, with little cellular attack against live microfilariae (generalized onchocerciasis). Clinical lesions correspond to infiltrates around dead or degenerating microfilariae surrounded by macrophages, eosinophils, and neutrophils [16]. As in the cornea, inflammation appears to be largely induced by *Wolbachia*endobacterial products [13]. In generalized onchocerciasis, the T helper cell type 1– and T helper cell type 2–dependent effector reactions are suppressed by a third arm of the T helper pathway, the T helper cell type 3, or T regulatory cell type 1 [17]. Antigen-specific T regulatory cell type 1 cells constitute a major source of interleukin 10, leading to a downregulation of the immune system that both prevents immune-mediated damage and facilitates parasite survival [13]. By contrast, patients with severe or hyperreactive skin lesions, such as lichenified onchodermatitis or sowda, often present with low microfilarial loads. Their lesions are due to repeated cycles of inflammation, eosinophil and macrophage infiltration, and destruction of live and dead microfilariae [18]. These different immune responses to the parasite and ensuing clinical presentation may be influenced by host genetic factors [19].

Onchocerciasis is also a systemic disease that is associated with musculoskeletal pain, reduced body mass index, and decreased work productivity. This may be due to the fact that microfilariae can invade many tissues and organs, and be found in blood and urine [5]. Involvement of heavy microfilarial infection is also suspected in the onset of epilepsy [20] and the hyposexual dwarfism known as Nakalanga syndrome [21]. A direct association between microfilarial load and excess mortality of the human host has been demonstrated recently [22] (Figure 3B).

## Epidemiological Patterns

In contrast with some soil-transmitted helminths and schistosomes, whose worm burdens typically peak in the young, age-specific patterns of *O. volvulus*infection show strong variation according to locality (microfilarial loads can increase, decrease, or plateau with age), and may differ markedly with host sex. Age- and sex-specific exposure, endocrine factors, and parasite-induced immunosuppression have been forwarded as possible explanations [23,24]. These patterns have implications for *O. volvulus*population biology and the design of control strategies.

The rationale behind the establishment of the OCP in savannah areas of 11 West African countries was based on the observation that there was a blinding savannah parasite strain, transmitted by savannah members of *S. damnosum*s.l., and a non-blinding forest strain, transmitted by forest members. Cross-experimental infections had indicated strong local adaptation and heterologous incompatibility, suggesting that the existence of *O. volvulus–S. damnosum*complexes could be responsible for the distinct distribution and severity of onchocercal blindness [25]. DNA-based methods confirmed an association between savannah and forest parasite types with, respectively, severe and mild ocular onchocerciasis [26]. In West African savannah, blindness prevalence correlates positively with intensity of infection in the community, a relationship rarely observed in West African forest [27]. The geographic distribution of severe and mild visual impairment is not, however, neatly confined to the savannah/forest divide. There are forest and forest–savannah mosaic areas with high blindness prevalence [28] and parasites distinct from those in West Africa, while in others, parasites genetically indistinguishable from West African savannah isolates are not associated with blindness [29,30]. The pathogenic differences of the various strains may be a function of their relative *Wolbachia*load [31].

## Disease Burden and Socioeconomic Consequences

The true burden of onchocerciasis has largely been underestimated. Excess mortality of the blind, particularly among males, may be considerable [32,33]. Even in sighted individuals, high microfilarial load can negatively affect a host's life expectancy [22]. Parasite-induced immunosuppression to specific and non-specific antigens [34], impairment of the ability to fend off infections and seroconvert successfully upon vaccinations [35], and manifestations such as epilepsy possibly due to heavy infection [20] may be partially responsible for excess mortality. It is also well known that onchodermatitis and epilepsy are associated with social stigmatization [36]. Onchocerciasis is deemed responsible for the annual loss of approximately 1 million disability-adjusted life-years—healthy life-years lost due to disability and mortality (more than half of them due to skin disease [37])—which greatly reduces income-generating capacity [38], incurs significant health expenditures, and exerts, overall, an immensely negative socioeconomic impact on the afflicted populations and their land use [39]. Although not the only cause of depopulation in some otherwise fertile West African valleys, onchocerciasis prevented resettlement of these arable lands [40]. The benefits accrued through onchocerciasis control programs should be measured not only in terms of blindness cases prevented and the cost-effectiveness of treatment [41,42], but also in terms of number of deaths averted.

## Onchocerciasis Control Strategies

The mainstay of onchocerciasis control is through antivectorial and antiparasitic measures. The former are directed against the black fly aquatic stages, and the latter against the microfilariae. As yet there is no effective macrofilaricidal drug that is safe for mass treatment. The OCP initially implemented weekly larviciding of vector breeding grounds, with the aim of interrupting transmission in the core OCP area. After achieving this, elimination of the parasite required abolishing vector sources for as long as microfilariae remain in human skin. This duration was deemed to be at least 14 years (considering the life expectancies of both adult worms and microfilariae) [43]. In some parts of the OCP area, children born after the initiation of vector control proved to be uninfected [44]. In 1987, Merck took the unprecedented decision to donate ivermectin (Mectizan), an effective and safe microfilaricide, for as long as necessary to eliminate onchocerciasis as a public health problem. Following this commitment, regular ivermectin distribution by mobile teams was introduced to complement vector control in some OCP areas, or as the sole intervention in others [45]. Ivermectin, given at the dose of 150 micrograms per kilogram of body weight, acts as a highly effective microfilaricide and inhibits microfilarial production by female worms for several months. Mass administration of ivermectin (to all those aged five years or older, excluding pregnant women and those breastfeeding a child younger than one week old) once or twice per year reduces morbidity and disability [46,47] and lowers transmission [48,49]. Given the high initial endemicity in some foci, annual regimes are not considered sufficient to achieve local elimination of parasite populations [50], unless very high therapeutic coverage (more than 80 percent of the total population) is achieved for at least 25 years without loss of treatment efficacy [51].

In Latin America, focal vector control was conducted in Guatemala with some degree of success against the local *S. ochraceum*s.l. vector [52], but was otherwise considered impractical. The Onchocerciasis Elimination Program for the Americas (OEPA) was initiated in 1993 as a regional partnership to eliminate all morbidity from onchocerciasis (and suppress its transmission wherever possible) in foci of the six affected Latin American countries [53]. OEPA's strategy is currently based on biannual mass ivermectin distribution, as it was considered that treatment every six months would have a greater impact on transmission [54] and female worm fecundity [55].

In 1995, the African Programme for Onchocerciasis Control (APOC) was launched in order to cover the remaining 19 African countries not protected under the OCP umbrella [56]. (Three of them, Kenya, Rwanda, and Mozambique, were found not to be endemic.) Since then, APOC's strategy has been based on annual ivermectin distribution. The levels of geographic (percentage of villages treated in an area) and therapeutic (percentage of population treated in a village) coverage achieved by mobile teams tended to be unsatisfactory, with little prospects of sustainability. Instead, APOC has implemented, with great success, the modality of community-directed treatment with ivermectin (CDTI), by which communities themselves appoint accountable local distributors [57]. By the end of 2005, 400 million treatments had been supplied by the Mectizan Donation Program, with an estimated 40 million people living in 90,000 African villages being treated by nearly 300,000 community distributors throughout APOC projects.

The average cost per person treated, including volunteers' time, is US$0.74, making CDTI highly cost-effective [9]. Besides, the cost per person treated as part of APOC (not including the value of Mectizan) is nearly 8.5 times cheaper than the cost per person protected, via vector control, under the OCP [42]. In addition, the CDTI strategy has empowered communities to such an extent that it is currently being used as a platform for integrating other, mainly chemotherapeutic community-based interventions (such as vitamin A supplementation and albendazole for lymphatic filariasis treatment). Integration with other control programs may help maintain high coverage levels as clinical symptoms of onchocerciasis subside [58]. However, in spite of its impressive achievements in terms of coverage, and the promising perspectives of combined community-directed interventions, APOC has to face serious challenges in terms of achieving its ultimate treatment goal of both long-term sustainability and substantial permanent impact.

In those areas where onchocerciasis and loiasis (caused by the filarial nematode *Loa loa*) are coendemic (mainly central Africa), ivermectin treatment for *O. volvulus*in individuals with high *L. loa*microfilaraemia can result in severe adverse events, including fatal encephalopathy [59]. This has represented an important setback to APOC's expansion. Geostatistical models are being developed to map the risk of heavy loiasis across Africa [60], and treatment protocols will be tested aimed to reduce *L. loa*microfilaraemia prior to ivermectin treatment.

Studies aimed at evaluating the sustainability of APOC-sponsored projects have also revealed that communities do not always support distributors adequately; the continued commitment of distributors is often maintained because of their involvement in other more “lucrative” activities, such as immunization. Lack of resources makes supervision difficult at the community and health facility levels, and many obstacles must yet be overcome to integrate CDTI successfully with other health activities [61].

These concerns raise questions as to how long APOC should last. When launched, it was anticipated that APOC's duration would be 12 years (1995 through 2007). Since then, a two-year phasing-out period has been added, and donors' support secured until 2010. Presently, no decisions regarding further extensions have been made, but, given the life cycles of the parasite and its vector, APOC's activities would likely need to be sustained for at least 20 years to have a significant and enduring impact [42].

## Need for Other Effective Compounds against *O. volvulus*

The increasing reliance of onchocerciasis control upon ivermectin alone, and the absence of a real breakthrough in vaccine development [62], have spurred research on other compounds. Moxidectin has emerged as a highly efficacious microfilaricide whose half-life in humans is longer than that of ivermectin [63]; it may therefore suppress adult worm fecundity for longer [63]. Its chemical structure is similar to that of ivermectin, and, in animal models, it does not seem to be truly macrofilaricidal [64].

Novel chemotherapeutic interventions could be based on the use of antibiotics against the endosymbiotic bacteria, as long-term depletion of *Wolbachia*impairs worm reproduction and survival [65]. Daily treatment with 100 milligrams of doxycycline for six weeks (or 200 milligrams daily for four weeks) leads to an interruption of embryogenesis that lasts for 18 months or more [66]. However, the prolonged duration of treatment, the various contraindications to antibiotics, and the risk of inducing resistance in other pathogens make it difficult to incorporate these regimens in mass chemotherapy programs. Research on the efficacy of other antibiotics and the shortest course of treatment that can effectively remove the bacteria permanently may help overcome some of these obstacles [67]. Alternatively, anti-*Wolbachia*therapy could be used to treat selectively those individuals identified as microfilaria-positive at the end of mass ivermectin distribution in order to “mop up” areas where parasite elimination is deemed feasible.

It is to be expected that the scaling up of all ivermectin-reliant control programs (previous OCP countries and those within APOC and OEPA) will impose selection pressures on the parasite genome. Although no confirmed case of ivermectin resistance has yet been identified, a phenotype of suboptimal response to the drug has been reported in localities in Ghana subjected to more than nine treatments [68]. This phenomenon appears to be explained not by loss of microfilaricidal efficacy, but by adult females resuming reproductive activity earlier than expected. Evidence of selection operating upon polymorphic loci (associated with ivermectin resistance in veterinary nematodes) has been documented by genetic analysis of worms obtained from patients who had received six or more annual doses in comparison to those who were ivermectin-naïve [69]. However, the definitive studies linking response phenotype to parasite genotype with increasing treatment doses have yet to be conducted. Mathematical models can help understand parasite population biology processes that influence rates of infection recrudescence [70,71] and the spread of alleles favored by ivermectin-induced selection.

## Modeling for Onchocerciasis Control

Onchocerciasis is one of the best examples in the history of parasitic control in which intervention strategies have been informed at all stages by computer simulation models. In particular, ONCHOSIM, a computer program for modeling onchocerciasis transmission and control, was developed under the sponsorship of OCP for West African savannah settings [72]. Other models pertain to transmission and control in forest areas and Latin American foci [73]. The key question of how long antifilarial treatment should be administered depends on the anticipated goals and the particular epidemiology of specific foci. If the objective is elimination of onchocerciasis as a public health problem, annual ivermectin administration in APOC countries will constitute a successful strategy once the levels of infection in the community are reduced below five to ten microfilariae per skin snip, but this is unlikely to interrupt transmission of *O. volvulus*in Africa [74]. Factors such as the intensity and seasonality of transmission, the *Onchocerca–Simulium*combination(s) present, the parasite distribution among hosts, the density-dependent processes operating upon the parasite's life cycle, and the interaction of all these with control interventions and their coverage will determine the stability of the host–parasite system and our ability (or inability) to push *O. volvulus*below possible transmission breakpoints [70,71,73].

## Conclusion

Neglect manifests itself in many guises. Financial and political commitment are required not only to support the control programs but also to fund the research necessary to provide the tools to enable parasite elimination. The spectacular success of the OCP has pushed onchocerciasis down to the bottom of the health research agenda at a time when consolidating its achievements and demonstrating long-term success in APOC and OEPA are of utmost importance. Priority should be given to the development of tools for improved diagnosis (detection of skin microfilariae will lose sensitivity as control progresses, and parasite antigen tests have proved elusive), efficacious killing of adult worms, early detection of potential loss of drug efficacy and associated parasite genetic changes, and better understanding of the impact of chemotherapeutic interventions upon the population biology of *O. volvulus*. At present, the prospect of indefinite ivermectin distribution risks the development of anthelmintic resistance and tempts public and donor fatigue.

## Supporting Information

Text S1 Translation of the Article into German (60 KB DOC).

Text S2 Translation of the Article into French (122 KB DOC).

Text S3 Translation of the Article into Spanish (100 KB DOC).

## Abbreviations

APOC: African Programme for Onchocerciasis Control
CDTI: community-directed treatment with ivermectin
OCP: Onchocerciasis Control Programme in West Africa
OEPA: Onchocerciasis Elimination Program for the Americas
s.l.: sensu lato (denotes a complex of simuliid sibling species)
