# Supplementary material for: River Blindness: A Success Story under Threat?
Source: PLoS Med. 2006 Sep 26;3(9):e371. doi: 10.1371/journal.pmed.0030371 (PMC1576321; doi:10.1371/journal.pmed.0030371)
Supplement: Text S1 — (60 KB DOC). [file pmed.0030371.sd001.doc]

Flussblindheit: eine Erfolgsgeschichte in Gefahr?
María-Gloria Basáñez, Sébastien D. S. Pion, Thomas S. Churcher, Lutz P. Breitling, Mark P. Little, Michel Boussinesq

Abkürzungen: APOC, African Programme for Onchocerciasis Control; CDTI, community-directed treatment with ivermectin; OCP, Onchocerciasis Control Programme in West Africa; OEPA, Onchocerciasis Elimination Program for the Americas; s.l., sensu lato.

Einleitung
„Die Erfolge dieses Programms inspirieren uns alle im Public Health-Bereich, große Träume zu träumen. Sie zeigen, dass wir ‚unmögliche' Ziele erreichen und das Leiden von Millionen der ärmsten Menschen der Welt mildern können. [...].“ Dies waren die Schlussworte des früheren Generaldirektors der Weltgesundheitsorganisation, Gro Harlem Brundtland, bei der Schlusszeremonie des Onchocerciasis Control Programme in Westafrika (OCP) im Dezember 2002 [1]. Der Erfolg des OCP ist so unbestreitbar und beispielhaft - 600.000 Blindheitsfälle wurden verhindert, 18 Millionen Kinder wurden in von Blindheitsrisiko befreiten Gebieten geboren und 25 Millionen Hektar Land für Wiederbesiedelung gesichert - , dass Flussblindheit derzeit als Krankheit der Vergangenheit betrachtet wird. Dabei wird jedoch übersehen, dass das OCP höchstens 1.200.000 km² und 30 Millionen Menschen in 11 Ländern erreichte, womit etwa 100 Millionen Menschen in Gebieten verbleiben, in denen immer noch  aktive Übertragung von  Flussblindheit vorkommt. Nach dem 28-jährigen Kampf des OCP haben wir vielleicht „eine Schlacht gewonnen“, aber eine sehr viel schwierigere Herausforderung liegt vor uns, bevor wir die Flussblindheit als besiegt ansehen können [2].

Ätiologie und Verbreitung
Die Flussblindheit (Onchocerciasis, Onchozerkose) des Menschen wird vom parasitären Nematoden Onchocerca volvulus, einer Filarienart, verursacht. Die erwachsenen Würmer (Makrofilarien) leben in Knoten im Unterhautgewebe und tieferen Konglomeraten, in denen befruchtete Weibchen im Verlauf von durchschnittlich etwa 10 Jahren Millionen von Mikrofilarien produzieren können, die für die mit der Infektion verbundenen Morbidität verantwortlich sind. Während der Blutmahlzeit von Kriebelmücken (Gattung Simulium; engl. black flies) aufgenommen, entwickeln sich die Mikrofilarien in den Mücken zu infektiösen (L3) Stadien, übertragbar auf andere Menschen (Abb. 1). Zahlreiche Simulium-Arten wurden mehr oder weniger der Übertragung von O. volvulus verdächtigt [3], und ihre relativen Rollen als Vektoren tragen zur Ausbildung unterschiedlicher Übertragungsmuster in verschiedenen endemischen Gebieten bei. In Afrika ist der S. damnosum sensu lato (s.l.)-Komplex, der etwa 60 Zytoformen umfasst, für mehr als 95% der weltweiten Onchozerkose-Fälle verantwortlich [3,4]. In Lateinamerika sind S. onchraceum s.l., S. exiguum s.l., S. metallicum s.l. bzw. S. guianense s.l. die Hauptüberträger, in Mexiko und Guatemala (ca. 360.000 Menschen mit Infektionsrisiko), Kolumbien und Ecuador (ca. 24.600), Nordvenezuela (ca. 104.500) bzw. Südvenezuela und Brasilien (ca. 20.000) [5,6].
O. volvulus, endemisch in 27 subsaharischen Ländern Afrikas und dem Jemen [7], wurde durch Sklavenhandel in sechs lateinamerikanische Länder eingeschleppt. Frühere Schätzungen lagen bei 18 Millionen Infizierten weltweit [7], 99% davon in Afrika. In der Folge wurde das wahre Ausmaß der Krankheit mit Rapid Epidemiological Mapping of Onchocerciasis (REMO; dt. schnelle epidemiologische Kartierung von Onchozerkose) geschätzt: In jedem Flussbecken werden Dörfer nach angemessenen Kriterien ausgewählt und Endemizitätslevel anhand der Prävalenz von Onchozerkose-Knoten in einer Kohorte von Erwachsenen erhoben [8]. Bis 2005 wurden mehr als 22.000 Dörfer (außerhalb des OCP-Gebiets) untersucht, und dies erlaubte die Identifizierung zahlreicher neuer Krankheitsherde (Abb. 2). Die dabei neu gefundenen infizierten Populationen und ihr demographisches Wachstum kompensieren sicherlich die Anzahl der durch das OCP verhinderten Fälle (es war damals geschätzt worden, dass etwa 3 Millionen Menschen infiziert waren [7]). Gegenwärtig wird geschätzt, dass etwa 37 Millionen Menschen mit O. volvulus infiziert und etwa 90 Millionen in Afrika von Infektion bedroht sind [9].

Klinische Manifestation und Pathogenese
Aufgrund der hohen Blindheitsprävalenz in Dörfern entlang schnell-fließender Flüsse, wo die Überträger brüten, ist die Onchozerkose besser bekannt unter dem Namen "Flussblindheit". Bis zu 500.000 Fälle schwerwiegender visueller Beeinträchtigung (einschließlich Gesichtsfeldreduktion) und 270.000 Fälle von Blindheit werden der Onchozerkose zugeschrieben [7], doch auch diese Zahlen unterschätzen sicherlich das wahre Ausmaß des Problems. Okulare Läsionen können alle Gewebe des Auges betreffen und reichen von Keratitis punctata und sklerosierender Keratitis (vorderer Augenabschnitt) bis zu Sehnervatrophie (hinterer Augenabschnitt). Für die Blindheitsinzidenz wurde vor kurzem bei Personen, die in der OCP-Kohorte nachverfolgt wurden, eine Assoziation mit der zurückliegenden Mikrofilarienlast gezeigt [10]. Dies bestätigte die progressive Verschlechterung der onchozerkalen Erkrankung des Auges mit Parasitenexposition (Abb. 3a). Ursprünglich wurden die Vorderkammerläsionen einer von Filarienprodukten ausgelösten Kaskade von Entzündungsprozessen zugeschrieben [11]. Eine neue Hypothese nimmt an, dass die zu zunehmender Kornea-Trübung führenden pro-inflammatorischen Vorgänge nicht nur vom Parasiten selbst, sondern auch von seinen kürzlich entdeckten endosymbiotischen Wolbachia-Bakterien stimuliert werden, die von sterbenden Mikrofilarien freigesetzt werden [12,13]. Im Gegensatz dazu könnte die Pathogenese der Netzhautläsionen, die trotz Parasitenbeseitigung nach Chemotherapie fortschreiten kann, auf Autoimmunprozessen beruhen, die durch Kreuzreaktivität zwischen dem O. volvulus-Antigen Ov39 und dem humanen Retina-Antigen hr44 ausgelöst werden [14].
Onchozerkose verursacht auch lästigen Juckreiz und Hautveränderungen, die von frühen, „reaktiven“ Läsionen - akuter papulöser Onchodermatitis (APOD), chronischer papulöser Onchodermatitis (CPOD) und lichenifizierter Onchodermatitis (LOD) - bis zu Spätfolgen wie Depigmentierung und Hautatrophie reichen [15]. Falls sie auf eine Extremität beschränkt bleibt, wird die LOD auch als „Sowda“ bezeichnet. In endemischen Gebieten präsentieren sich die meisten Betroffenen trotz hoher Mikrofilarienlast in der Haut mit subklinischer oder intermittierender Dermatitis, einer APOD entsprechend, mit geringer zellulärer Reaktion gegen lebende Mikrofilarien („generalisierte Onchozerkose“). Klinische Läsionen entsprechen Infiltraten aus Makrophagen, Eosinophilen und Neutrophilen, die tote oder degenerierende Mikrofilarien umgeben [16]. Die Entzündung scheint, wie in der Kornea, weitgehend von endobakteriellen Wolbachia-Produkten induziert zu werden [13]. Im Falle generalisierter Onchozerkose werden Th1- und Th2-abhängige Effektorreaktionen von einem dritten Arm des T-Helfer-Systems unterdrückt, den Th3- oder regulatorischen T-Zellen Typ 1 (Tr-1) [17]. Antigen-spezifische Tr-1-Zellen stellen eine Hauptquelle von IL-10 dar und führen zu einer Herunterregulierung des Immunsystems, die sowohl immunreaktionsbedingte Schäden verhindert als auch das Überleben der Parasiten erleichtert [13]. Im Gegensatz dazu zeigen Patienten mit schweren oder „hyperreaktiven“ Hautläsionen wie LOD oder Sowda oft niedrige Mikrofilarienlasten. Ihre Läsionen beruhen auf wiederholten Zyklen von Entzündung, Infiltration von Eosinophilen und Makrophagen und Zerstörung von lebenden und abgestorbenen Mikrofilarien [18]. Diese unterschiedlichen Immunreaktionen gegen den Parasiten und die daraus resultierenden klinischen Bilder können von genetischen Faktoren des Wirts beeinflusst werden [19].
Onchozerkose ist auch eine systemische Krankheit, die mit muskuloskeletalem Schmerz, vermindertem Body Mass Index und herabgesetzter Arbeitsproduktivität einhergeht. Dies könnte damit zusammenhängen, dass Mikrofiliarien zahlreiche Gewebe und Organe infiltrieren und in Blut und Urin gefunden werden können [5]. Es wird ebenso vermutet, dass schwere mikrofilarielle Infektion bei der Manifestation von Epilepsie [20] und der als „Nakalanga-Syndrom“ bekannten Form des hyposexuellen Minderwuchses [21] eine Rolle spielt. Eine direkte Assoziation von Mikrofilarienlast und Exzessmortalität des menschlichen Wirts wurde kürzlich gezeigt [22] (Abb. 3b).

Epidemiologische Muster
Im Gegensatz zu verschiedenen über den Boden übertragenen Helminthen und Schistosomen, bei denen die Wurmlast typischerweise einen Peak bei jungen Menschen aufweist, zeigen die altersspezifischen Verteilungsmuster der O. volvulus-Infektion eine starke Variation zwischen verschiedenen Orten (die Mikrofilarienlast kann mit dem Alter zunehmen, abnehmen oder ein Plateau erreichen) und kann ausgeprägte Geschlechtsunterschiede aufweisen. Alters- und geschlechtsspezifische Exposition, endokrine Faktoren und Parasiten-induzierte Immunsuppression wurden als mögliche Erklärungen vorgeschlagen [23,24]. Die verschiedenen Muster haben Bedeutung für die Populationsbiologie von O. volvulus sowie das Design von Kontrollstrategien.
Die Grundlage für die Durchführung des OCP in Savannengebieten von 11 westafrikanischen Ländern fußte auf der Beobachtung, dass es einen „blind machenden“ Savannen-Stamm des Parasiten gab, übertragen von Savannen-Vertretern von Simulium damnosum s.l., und einen „nicht blind machenden“ Wald-Stamm, übertragen von Kriebelmücken des Regenwalds. Kreuzinfektionsexperimente hatten starke lokale Adaption und heterologe Inkompatibilität gezeigt und nahegelegt, dass die Existenz von O. volvulus-S. damnosum-Komplexen für die differenzierte Verbreitung und Schwere der Flussblindheit verantwortlich sein könnte [25]. DNA-basierte Methoden bestätigten die Assoziation von parasitären Savannen- bzw. Wald-Typen mit schwerer bzw. leichter okulärer Onchozerkose [26]. In der westafrikanischen Savanne ist die Blindheitsprävalenz positiv mit der Infektionsintensität in der Bevölkerung korreliert, ein Zusammenhang, der im westafrikanischen Regenwald selten beobachtet wird [27]. Die geographische Verbreitung von schwerer oder leichter Beeinträchtigung des Sehvermögens folgt jedoch nicht exakt der Savannen/Wald-Grenze. Es gibt Wald- und Wald-Savannen-Mosaikgebiete mit hoher Prävalenz von Blindheit [28] und Erregern, die sich von denen Westafrikas unterscheiden, während in anderen Gebieten Parasiten, die von den westafrikanischen Savannen-Isolaten genetisch nicht zu unterscheiden sind, nicht mit Blindheit assoziiert sind [29,30]. Die pathogenetischen Unterschiede der verschiedenen Stämme beruhen möglicherweise auf ihrer relativen Wolbachia-Last [31].

Krankheitsbelastung und sozioökonomische Auswirkungen
Die wirkliche Belastung durch Onchozerkose ist in hohem Maße unterschätzt worden. Die Exzessmortalität der Blinden, insbesondere unter Männern, kann beträchtlich sein [32,33]. Selbst bei sehenden Personen kann hohe Mikrofilarienlast die Lebenserwartung des Wirtes negativ beeinflussen [22]. Parasiten-induzierte Immunsuppression gegenüber spezifischen und unspezifischen Antigenen [34], Beeinträchtigung der Fähigkeit zur Infektionsabwehr und erfolgreicher Serokonversion nach Vakkzination [35] und Manifestationen wie Epilepsie, möglicherweise durch schwere Infektionen ausgelöst [20], könnten teilweise für die Exzessmortalität verantwortlich sein. Es ist auch bekannt, dass Onchodermatitis und Epilepsie mit sozialer Stigmatisierung verbunden sind [36]. Onchozerkose wird für den Verlust von jährlich  etwa einer Million disability-adjusted life-years (DALYs) - aufgrund von Behinderung und Mortalität verlorenen Lebensjahren - verantwortlich erachtet, wobei mehr als die Hälfte davon auf dermale Manifestationen zurückzuführen sind [37]. Dies reduziert die Kapazität, Einkommen zu generieren, beträchtlich [38], verursacht hohe Gesundheitskosten und hat alles in allem ausgesprochen negative sozioökonomische Folgen auf die betroffenen Populationen und ihre Landnutzung [39]. Obwohl sie nicht der einzige Grund für die Entvölkerung mancher ansonsten fruchtbarer Flusstäler Westafrikas war, verhinderte die Onchozerkose die Wiederbesiedlung dieser urbaren Gegenden [40]. Der aus Onchozerkose-Kontrollprogrammen erwachsene Nutzen sollte nicht nur an den verhinderten Erblindungen und der Wirtschaftlichkeit der Behandlung gemessen werden [41,42], sondern auch an der Zahl der abgewendeten Todesfälle.

Strategien zur Kontrolle der Onchozerkose
Die Onchozerkose-Kontrolle stützt sich hauptsächlich auf antivektorielle und antiparasitäre Maßnahmen. Erstere richten sich gegen die im Wasser lebenden Stadien der Kriebelmücken, letztere gegen die Mikrofilarien. Bislang gibt es kein effektives, zur Massenbehandlung geeignetes Makrofilarizid. Zu Anfang implementierte das OCP wöchentliche Larvizid-Ausbringungen an Vektorbrutstätten, um die Übertragung im OCP-Kerngebiet zu unterbrechen. Nachdem dies erreicht worden war, bedurfte es zur Eliminierung des Parasiten der Beseitigung der Vektorquellen für so lange, wie Mikrofilarien in der Haut des infizierten Wirts vorhanden sind. Diese Zeitspanne wurde auf wenigstens 14 Jahre geschätzt (in Anbetracht der Lebenserwartung von Adultwürmern und Mikrofilarien) [43]. In einigen Teilen des OCP-Gebietes waren Kinder, die nach Initiierung der Vektorkontrolle geboren wurden, tatsächlich nicht infiziert [44]. Im Jahr 1987 trafen Merck die beispiellose Entscheidung, Ivermectin (Mectizan), ein wirksames und sicheres Mikrofilarizid, so lange kostenlos zur Verfügung zu stellen, wie zur Beseitigung der Onchozerkose als Public Health-Problem notwendig sein sollte. In der Folge dieses Engagements wurde in einigen OCP-Gebieten regelmäßige Ivermectin-Verteilung durch mobile Teams als Zusatzmaßnahme zur Vektorkontrolle eingeführt, in anderen Gebieten als alleinige Intervention [45]. In einer Dosierung von 150 µg/kg Körpergewicht ist Ivermectin ein hoch wirksames Mikrofilarizid und inhibiert die Mikrofilarienproduktion der weiblichen Würmer für mehrere Monate. Massenanwendung von Ivermectin (bei allen Personen ab 5 Jahren, ausschließlich Schwangerer oder ein unter einer Woche altes Kind stillender Frauen), ein- oder zweimal pro Jahr, reduziert Morbidität und Invalidität [46,47] und vermindert die Übertragung [48,49]. Aufgrund der sehr hohen Ausgangsendemizität in manchen Foki werden jährliche Regime als nicht ausreichend zur lokalen Eliminierung von Parasitenpopulationen erachtet [50], wenn nicht eine sehr hohe therapeutische Abdeckung (>80% der Gesamtpopulation) für mindestens 25 Jahre und ohne Abnahme der Behandlungswirksamkeit erreicht werden kann [51].
In Lateinamerika wurde in Guatemala mit einigem Erfolg fokale Vektorkontrolle gegen den Vektor S. ochraceum s.l. durchgeführt [52], ansonsten jedoch als nicht praktikabel angesehen. Das Onchocerciasis Elimination Program for the Americas (OEPA; dt. Programm zur Eliminierung der Onchozerkose in den Amerikas) wurde 1993 als regionale Partnerschaft mit dem Ziel ins Leben gerufen, die gesamte Onchozerkose-Morbidität in den Foki der sechs betroffenen lateinamerikanischen Ländern zu beseitigen (und ihre Übertragung wo immer möglich zu unterdrücken) [53]. Die Strategie des OEPA basiert gegenwärtig auf halbjährlicher Ivermectin-Massenverteilung, da angenommen wurde, dass 6-monatliche Behandlung eine stärkere Wirkung auf die Übertragung [54] und die Fekundität der weiblichen Würmer hätte [55].
Im Jahre 1995 wurde das African Programme for Onchocerciasis Control (APOC; dt. Afrikanisches Programm zur Onchozerkose-Kontrolle) gestartet, um die 19 verbliebenen und nicht vom OCP erfassten afrikanischen Länder abzudecken [56]. (Es stellte sich heraus, dass drei davon, Kenia, Ruanda und Mosambik, nicht endemisch waren.) Seitdem stützt sich die APOC-Strategie auf jährliche Ivermectin-Verteilung. Der durch mobile Teams erreichte Grad geographischer (% behandelte Dörfer in einem Gebiet) und therapeutischer Abdeckung (% behandelte Bevölkerung in einem Dorf) war eher unbefriedigend und mit wenig Aussicht auf Nachhaltigkeit. Mit großem Erfolg führte das APOC jedoch die Modalität des community-directed treatment with ivermectin (CDTI; dt. gemeindegestützte Ivermectin-Behandlung) ein [57], wobei die Gemeinden selbst verantwortliche örtliche Verteiler ernennen. Bis Ende 2005 sind 400 Millionen Behandlungen vom Mectizan Donation Program ausgegeben und schätzungsweise 40 Millionen Menschen in 90.000 afrikanischen Dörfern von etwa 300.000 lokalen Verteilern im Rahmen der APOC-Projekte behandelt worden.
Die durchschnittlichen Kosten pro behandelter Person, einschließlich der Freiwilligenarbeit, betragen US$ 0,74, womit CDTI sehr kosteneffizient ist [9]. Des Weiteren sind die Kosten für eine unter APOC behandelte Person (ohne den Wert von Mectizan zu berücksichtigen) nahezu 8,5-mal billiger als die Kosten für eine durch Vektorkontrolle unter OCP geschützte Person [42]. Zusätzlich hat die CDTI-Strategie Dorfgemeinschaften derart befähigt, dass sie gegenwärtig als Plattform für weitere hauptsächlich chemotherapeutische gemeindebasierte Maßnahmen (wie Vitamin-A-Programme oder Albendazol zur Behandlung der lymphatischen Filariasis) genutzt wird. Die Integration mit anderen Kontrollprogrammen dürfte helfen, eine hohe Abdeckung zu erhalten, wenn klinische Onchozerkose-Symptome abnehmen [58]. Jedoch trotz seiner beeindruckenden Erfolge hinsichtlich Abdeckung und den vielversprechenden Aussichten von kombinierten „community-directed“ Interventionen, steht APOC vor ernsthaften Herausforderungen, um sein letztlich angestrebtes Behandlungsziel, langfristige Aufrechterhaltbarkeit und dauerhafte Wirkung, zu erreichen.
In Gebieten, in denen Onchozerkose und Loiasis (verursacht durch die Filarie Loa loa) co-endemisch sind (hauptsächlich Zentralafrika), kann die Ivermectin-Behandlung für O. volvulus bei Personen mit hoher L. loa-Mikrofilariämie schwere Nebenwirkungen haben, einschließlich tödlicher Enzephalopathie [59]. Dies bedeutete einen schweren Rückschlag für die Ausweitung von APOC. Geostatistische Modelle sind in Entwicklung, um das Risiko schwerer Loiasis Afrika-weit zu kartieren [60], und Behandlungsprotokolle werden untersucht werden mit dem Ziel, L. loa-Mikrofilariämie vor Ivermectin-Behandlung zu senken. 
Studien zur Evaluierung der Aufrechterhaltbarkeit APOC-geförderter Projekte zeigten auch, dass Gemeinden die Verteiler nicht immer angemessen unterstützen; ihr Engagement dauerte oftmals nur aufgrund der Beteiligung an anderen, „lukrativeren“ Aktivitäten wie Impfungen an. Der Mangel an Mitteln erschwert die Supervision auf Ebene der Gemeinden und Gesundheitseinrichtungen, und viele Hindernisse müssen noch bewältigt werden, um CDTI erfolgreich in andere Gesundheitsprojekte zu integrieren [61].
Diese Probleme führen zu der Frage, wie lange APOC unterhalten werden sollte. Zu Beginn wurde für das APOC eine Laufzeit von 12 Jahren (1995 bis 2007) angenommen. Seitdem wurde eine zweijährige Auslaufzeit hinzugefügt und Geberunterstützung bis 2010 gesichert. Zum jetzigen Zeitpunkt sind keine Entscheidungen hinsichtlich zusätzlicher Erweiterungen getroffen worden, doch müssten die APOC-Aktivitäten - unter Berücksichtigung der Lebenzyklen von Parasit und Vektor - wahrscheinlich für wenigsten 20 Jahre weitergeführt werden, um eine signifikante und anhaltende Wirkung zu erzielen [42].

Bedarf an anderen effektiven Wirkstoffen gegen O. volvulus
Die zunehmende Abhängigkeit der Onchozerkose-Bekämpfung von Ivermectin allein und das Fehlen eines echten Durchbruchs in der Impfstoffentwicklung [62], haben die Erforschung anderer Wirkstoffe angetrieben. Moxidectin hat sich als hochwirksames  Mikrofilarizid herausgestellt, dessen Halbwertszeit im Menschen länger als die von Ivermectin ist [63]; es könnte daher die Adultwurmfekundität  länger unterdrücken [64]. Seine chemische Struktur ähnelt der von Ivermectin und in Tiermodellen scheint es nicht wirklich makrofilarizid zu sein [64].
Neue chemotherapeutische Interventionen könnten auf der Anwendung von Antibiotika gegen die endosymbiotischen Bakterien basieren, da die langfristige Depletion von Wolbachia die Reproduktion und das Überleben der Würmer beeinträchtigt [65]. Tägliche Behandlung mit 100 mg Doxycyclin über einen Zeitraum von 6 Wochen (oder 200 mg tgl. über 4 Wochen) führt zu einer Unterbrechung der Embryogenese, die ≥ 18 Monate anhält [66]. Die lange Behandlungsdauer, die verschiedenen Kontraindikationen gegen Antibiotika sowie die Gefahr, Resistenzen bei anderen Krankheitserregern herbeizuführen, erschweren es jedoch, diese Behandlungen in Massenchemotherapieprogrammen anzuwenden. Forschungsarbeit hinsichtlich der Wirksamkeit weiterer Antibiotika und der kürzesten Behandlungsdauer, die eine anhaltende Beseitigung der Bakterien erzielt, kann möglicherweise einige dieser Hindernisse überwinden helfen [67]. Alternativ könnte die Behandlung gegen Wolbachia benutzt werden, um selektiv solche Personen zu behandeln, die nach Ivermectin-Massenverteilung noch als mikrofilarämisch identifiziert werden, um Gebiete endgültig zu säubern, in denen die Elimination des Erregers für möglich gehalten wird.
Es muss erwartet werden, dass die Ausweitung der Ivermectin nutzenden Kontrollprogramme (frühere OCP-Länder, APOC- und OEPA-Gebiete) Selektionsdruck auf das Parasitengenom ausüben wird. Zwar ist bisher kein bestätigter Fall von Ivermectin-Resistenz identifiziert worden, doch wurde von einem Phänotyp sub-optimaler Medikamentenempfindlichkeit in Gebieten mit mehr als 9 Behandlungsrunden in Ghana berichtet [68]. Dieses Phänomen scheint nicht auf einem Verlust mikrofilarizider Wirksamkeit zu beruhen, sondern auf einer unerwartet frühen Wiederaufnahme der Reproduktion durch die adulten Weibchen. Hinweise für auf polymorphe Loci wirkende Selektion (assoziiert mit Ivermectin-Resistenz bei Nematoden in Tieren) wurde durch die vergleichende genetische Analyse von Würmern aus Ivermectin-naiven Patienten und solchen, die 6 oder mehr jährliche Behandlungen erhalten hatten, gezeigt [69]. Definitive Studien über den Zusammenhang zwischen dem Empfindlichkeitsphänotyp und Genotyp des Parasiten in Abhängigkeit von steigenden Behandlungsdosen müssen jedoch erst noch durchgeführt werden. Mathematische Modelle können helfen, parasitäre populationsbiologische Prozesse zu verstehen, welche die Infektionsrekrudeszenz [70,71] und die Verbreitung von Allelen beeinflussen, die durch Ivermectin-bedingte Selektion bevorteilt werden.

Modellierung für die Onchozerkose-Bekämpfung
Onchozerkose ist eines der besten Beispiele in der Geschichte der Parasitenbekämpfung, in dem Interventionsstrategien in allen Stadien durch Computersimulationen unterstützt wurden. Insbesondere wurde unter Schirmherrschaft von OCP für die westafrikanischen Savannen-Bedingungen ONCHOSIM entwickelt [72]. Andere Modelle beziehen sich auf Übertragung und Bekämpfung in Waldgebieten und lateinamerikanischen Foki [73]. Die Schlüsselfrage, wie lange antifilarielle Behandlung durchgeführt werden sollte, hängt von den angestrebten Zielen und der besonderen Epidemiologie spezifischer Herde ab. Falls das Ziel die Beseitigung von Onchozerkose als Public Health-Problem ist, wird jährliche Ivermectin-Verabreichung in APOC-Ländern eine erfolgreiche Strategie darstellen, sobald die Infektionslevel in der Bevölkerung unter 5-10 Mikrofilarien pro Skin-Snip (eine Art diagnostischer Hautbiopsie) gesenkt sind, doch wird dies kaum zu einer Unterbrechung der Übertragung von O. volvulus in Afrika führen [74]. Faktoren wie die Intensität und Saisonalität der Übertragung, die vorhandenen Onchocerca-Simulium-Kombination(en), die Parasitenverteilung unter den Wirten, die auf den Parasitenzyklus wirkenden dichteabhängigen Prozesse und die Wechselwirkungen all dieser Faktoren mit den Bekämpfungsmaßnahmen und ihrer Reichweite, werden über die Stabilität des Wirt-Parasiten-Systems entscheiden und damit auch  über unsere Fähigkeit (oder Unfähigkeit), O. volvulus unter mögliche „transmission breakpoints“ (Durchseuchungslevel, unterhalb derer die Parasitenübertragung gegen Null strebt) zu drücken [70,71,73].

Abschließende Bemerkungen
Vernachlässigung - „Neglect“ - zeigt sich in vielerlei Gestalt. Finanzielles und politisches Engagement werden nicht nur zur Unterstützung der Bekämpfungsprogramme benötigt, sondern auch um die Erforschung von Instrumenten zu fördern, welche die Parasitenelimination ermöglichen. Der spektakuläre Erfolg des OCP hat die Onchozerkose auf der Tagesordnung der Gesundheitsforschung zu einem Zeitpunkt nach hinten gerückt, wo die Absicherung seiner Errungenschaften und der Nachweis von langfristigen Erfolgen in APOC und OEPA von größter Bedeutung sind. Priorität genießen sollte die Entwicklung verbesserter Methoden für die Diagnostik (die Detektion von Mikrofilarien in der Haut wird mit fortschreitender Onchozerkose-Bekämpfung an Sensitivität verlieren und Parasitenantigen-Tests haben sich als unzuverlässig erwiesen), für die effiziente Abtötung der Adultwürmer, Früherkennung potentieller Abnahme der Medikamentenwirksamkeit und damit verbundener genetischer Veränderungen der Parasiten sowie ein besseres Verständnis der Auswirkungen chemotherapeutischer Interventionen auf die Populationsbiologie von O. volvulus. Im Augenblick wird mit der Aussicht auf unbegrenzte Ivermectinverteilung nicht nur die Entwicklung von Anthelmintika-Resistenzen riskiert, sondern auch die Ermüdung von Öffentlichkeit und Geberorganisationen. 

Danksagung
Die hier diskutierten Ideen haben profitiert von der langjährigen Unterstützung der Autoren durch Organisationen wie dem Wellcome Trust (MGB), dem Medical Research Council UK (MGB, TSC), der Fondations pour la Recherche Medicale und Singer-Polignac, Frankreich (SDSP), der River Blindness Foundation, und dem UNICEF/UNDP/World Bank/WHO Special Programme for Research and Training in Tropical Diseases (TDR) (MB).

Literaturliste 
1. http://www.who.int/dg/speeches/2002/Ouagadougou/en 
2. Hopkins AD (2005) Ivermectin and onchocerciasis: Is it all solved? Eye 19: 1057–1066.
3. Crosskey RW (1990) The natural history of blackflies. Chichester: John Wiley & Sons. 711 p.
4. Crosskey RW, Howard TM (2004) A revised taxonomic and geographical inventory of world blackflies (Diptera : Simuliidae). London: The Natural History Museum. Abrufbar unter: http://www.nhm.ac.uk/research-curation/projects/blackflies. Zugegriffen am 24. April 2006.
5. Bradley JE, Whitworth J, Basáñez M-G (2005) Onchocerciasis. In: Cox FEG, Wakelin D, Gillespie SH, Despommier DD. Parasitology, Topley and Wilson's microbiology and microbial infections. 10th edition. London: Hodder Arnold. pp. 781–801.
6. World Health Organization (2005) Onchocerciasis (river blindness). Weekly epidemiological record 80: 257–260. Abrufbar unter: http://www.who.int/wer/2005/wer8030.pdf. Zugegriffen am 26. Juli 2006. 
7. World Health Organization (1995) Onchocerciasis and its control. Report of a WHO expert committee on onchocerciasis control. WHO Technical Report Series, number 852. Geneva: World Health Organization. 110 p.
8. Ngoumou P, Walsh JF, Macé JM (1994) A rapid mapping technique for the prevalence and distribution of onchocerciasis: A Cameroon case study. Ann Trop Med Parasitol 88: 463–474.
9. African Programme for Onchocerciasis Control [APOC] (2005) Final communiqué of the 11th session of the Joint Action Forum (JAF) of APOC, Paris, France, 6–9 December 2005. Ouagadougou (Burkina Faso): APOC.
10. Little MP, Basáñez M-G, Breitling LP, Boatin BA, Alley ES (2004) Incidence of blindness during the entire duration of the Onchocerciasis Control Programme in western Africa, 1971–2002. J Infect Dis 189: 1932–1941.
11. Hall LR, Pearlman E (1999) Pathogenesis of onchocercal keratitis (river blindness). Clin Microbiol Rev 12: 445–453.
12. Saint-André A, Blackwell NM, Hall LR, Hoerauf A, Brattig NW, et al. (2002) The role of endosymbiotic Wolbachia bacteria in the pathogenesis of river blindness. Science 295: 1892–1895.
13. Brattig NW (2004) Pathogenesis and host responses in human onchocerciasis: Impact of Onchocerca filariae and Wolbachia endobacteria. Microbes Infect 6: 113–128.
14. McKechnie NM, Gürr W, Yamada H, Copland D, Braun G (2002) Antigenic mimicry: Onchocerca volvulus antigen-specific T cells and ocular inflammation. Invest Ophthalmol Vis Sci 43: 411–418.
15. Murdoch ME, Hay RJ, Mackenzie CD, Williams JF, Ghalib HW, et al. (1993) A clinical classification and grading system of the cutaneous changes in onchocerciasis. Br J Dermatol 129: 260–269.
16. Pearlman E, Garhart CA, Grand DJ, Diaconu E, Strine ER, et al. (1999) Temporal recruitment of neutrophils and eosinophils to the skin in a murine model for onchocercal dermatitis. Am J Trop Med Hyg 61: 14–18.
17. Hoerauf A, Brattig N (2002) Resistance and susceptibility in human onchocerciasis—Beyond Th1 vs. Th2. Trends Parasitol 18: 25–31.
18. Ali MM, Baraka OZ, AbdelRahman SI, Sulaiman SM, Williams JF, et al. (2003) Immune responses directed against microfilariae correlate with severity of clinical onchodermatitis and treatment history. J Infect Dis 187: 714–717.
19. Meyer CG, Gallin M, Erttmann KD, Brattig N, Schnittger L, et al. (1994) HLA-D alleles associated with generalized disease, localized disease, and putative immunity in Onchocerca volvulus infection. Proc Natl Acad Sci U S A 91: 7515–7519.
20. Boussinesq M, Pion SD, Demanga-Ngangue, Kamgno J (2002) Relationship between onchocerciasis and epilepsy: A matched case-control study in the Mbam Valley, Republic of Cameroon. Trans R Soc Trop Med Hyg 96: 537–541.
21. Kipp W, Burnham G, Bamuhiiga J, Leichsenring M (1996) The Nakalanga syndrome in Kabarole district, Western Uganda. Am J Trop Med Hyg 54: 80–83.
22. Little MP, Breitling LP, Basáñez M-G, Alley ES, Boatin BA (2004) Association between microfilarial load and excess mortality in human onchocerciasis: An epidemiological study. Lancet 363: 1514–1521.
23. Filipe JA, Boussinesq M, Renz A, Collins RC, Vivas-Martinez S, et al. (2005) Human infection patterns and heterogeneous exposure in river blindness. Proc Natl Acad Sci U S A 102: 15265–15270.
24. Duerr HP, Dietz K, Schulz-Key H, Büttner DW, Eichner M (2003) Density-dependent parasite establishment suggests infection-associated immunosuppression as an important mechanism for parasite density regulation in onchocerciasis. Trans R Soc Trop Med Hyg 97: 242−250.
25. Duke BO, Lewis DJ, Moore PJ (1966) Onchocerca-Simulium complexes. I. Transmission of forest and Sudan-savanna strains of Onchocerca volvulus, from Cameroon, by Simulium damnosum from various West African bioclimatic zones. Ann Trop Med Parasitol 60: 318–336.
26. Zimmerman PA, Dadzie KY, De Sole G, Remme J, Alley ES, et al. (1992) Onchocerca volvulus DNA probe classification correlates with epidemiologic patterns of blindness. J Infect Dis 165: 964–968.
27. Dadzie KY, Remme J, Rolland A, Thylefors B (1989) Ocular onchocerciasis and intensity of infection in the community. II. West African rainforest foci of the vector Simulium yahense. Trop Med Parasitol 40: 348–354.
28. Kayembe DL, Kasonga D L, Kayembe PK, Mwanza JC, Boussinesq M (2003) Profile of eye lesions and vision loss: A cross-sectional study in Lusambo, a forest-savanna area hyperendemic for onchocerciasis in the Democratic Republic of Congo. Trop Med Int Health 8: 83–89.
29. Fischer P, Bamuhiiga J, Kilian AH, Büttner DW (1996) Strain differentiation of Onchocerca volvulus from Uganda using DNA probes. Parasitology 112: 401–408.
30. Higazi TB, Katholi CR, Mahmoud BM, Baraka OZ, Mukhtar MM, et al. (2001) Onchocerca volvulus: Genetic diversity of parasite isolates from Sudan. Exp Parasitol 97: 24–34.
31. Higazi TB, Filiano A, Katholi CR, Dadzie Y, Remme JH, et al. (2005) Wolbachia endosymbiont levels in severe and mild strains of Onchocerca volvulus. Mol Biochem Parasitol 141: 109–112.
32. Prost A (1986) The burden of blindness in adult males in the savanna villages of West Africa exposed to onchocerciasis. Trans R Soc Trop Med Hyg 80: 525–527.
33. Pion SD, Kamgno J, Demanga-Ngangue, Boussinesq M (2002) Excess mortality associated with blindness in the onchocerciasis focus of the Mbam Valley, Cameroon. Ann Trop Med Parasitol 96: 181–189.
34. Stewart GR, Boussinesq M, Coulson T, Elson L, Nutman T, et al. (1999) Onchocerciasis modulates the immune response to mycobacterial antigens. Clin Exp Immunol 117: 517–523.
35. Cooper PJ, Espinel I, Paredes W, Guderian RH, Nutman TB (1998) Impaired tetanus-specific cellular and humoral responses following tetanus vaccination in human onchocerciasis: A probable role for interleukin-10. J Infect Dis 178: 1133–1138.
36. Vlassoff C, Weiss M, Ovuga EB, Eneanya C, Newel PT, et al. (2000) Gender and the stigma of onchocercal skin disease in Africa. Soc Sci Med 50: 1353–1368.
37. Remme JH (2004) Research for control: The onchocerciasis experience. Trop Med Int Health 9: 243–254.
38. Oladepo O, Brieger WR, Otusanya S, Kale OO, Offiong S, et al. (1997) Farm land size and onchocerciasis status of peasant farmers in south-western Nigeria. Trop Med Int Health 2: 334–340.
39. Evans TG (1995) Socioeconomic consequences of blinding onchocerciasis. Bull World Health Organ 73: 495–506.
40. Hervouët JP, Prost A (1979) Organisation de l'espace et épidémiologie de l'onchocercose. In: Maîtrise de l'espace agraire et développement en Afrique tropicale. Mémoires ORSTOM, number 89. Paris: ORSTOM. pp. 179–190.
41. Benton B (1998) Economic impact of onchocerciasis control through the African Programme for Onchocerciasis Control: An overview. Ann Trop Med Parasitol 92 (Suppl): S33–S39.
42. Waters HR, Rehwinkel JA, Burnham G (2004) Economic evaluation of Mectizan distribution. Trop Med Int Health 9 (Suppl): A16–A25.
43. Hougard JM, Alley ES, Yaméogo L, Dadzie KY, Boatin BA (2001) Eliminating onchocerciasis after 14 years of vector control: A proved strategy. J Infect Dis 184: 497−503.
44. Ba O, Karam M, Remme J, Zerbo G (1987) Place des enfants dans l'évaluation du programme de lutte contre l'onchocercose en Afrique de l'ouest [Role of children in the evaluation of the Onchocerciasis Control Program in West Africa]. Trop Med Parasitol 38: 137–142.
45. Molyneux DH (1995) Onchocerciasis control in West Africa: Current status and future of the Onchocerciasis Control Programme. Parasitol Today 11: 399–402.
46. Ejere H, Schwartz E, Wormald R (2001) Ivermectin for onchocercal eye disease (river blindness). Cochrane Database Syst Review: 2001: CD002219. 
47. Tielsch JM, Beeche A (2004) Impact of ivermectin on illness and disability associated with onchocerciasis. Trop Med Int Health 9 (Suppl): A45–A56.
48. Boussinesq M, Prod'hon J, Chippaux JP (1997) Onchocerca volvulus: Striking decrease in transmission in the Vina valley (Cameroon) after eight annual large scale ivermectin treatments. Trans R Soc Trop Med Hyg 91: 82−86.
49. Collins RC, Gonzalez-Peralta C, Castro J, Zea-Flores G, Cupp MS, et al. (1992) Ivermectin: Reduction in prevalence and infection intensity of Onchocerca volvulus following biannual treatments in five Guatemalan communities. Am J Trop Med Hyg 47: 156−169.
50. Borsboom GJ, Boatin BA, Nagelkerke NJ, Agoua H, Akpoboua KL, et al. (2003) Impact of ivermectin on onchocerciasis transmission: Assessing the empirical evidence that repeated ivermectin mass treatments may lead to elimination/eradication in West Africa. Filaria J 2: 8.
51. Winnen M, Plaisier AP, Alley ES, Nagelkerke NJ, van Oortmarssen G, et al. (2002) Can ivermectin mass treatments eliminate onchocerciasis in Africa? Bull World Health Organ 80: 384–390.
52. Ochoa JO, Castro JC, Barrios VM, Juarez EL, Tada I (1997) Successful control of onchocerciasis vectors in San Vicente Pacaya, Guatemala, 1984–1989. Ann Trop Med Parasitol 91: 471−479.
53. Richards FO, Boatin B, Sauerbrey M, Sékétéli A (2004) Control of onchocerciasis today: Status and challenges. Trends Parasitol 17: 558–563.
54. Cupp EW, Ochoa JO, Collins RC, Cupp MS, Gonzalez-Peralta C, et al. (1992) The effects of repetitive community-wide ivermectin treatment on transmission of Onchocerca volvulus in Guatemala. Am J Trop Med Hyg 47: 170−180.
55. Duke BO, Zea-Flores G, Castro J, Cupp EW, Muñoz B (1991) Comparison of the effects of a single dose and of four six monthly doses of ivermectin on adult Onchocerca volvulus. Am J Trop Med Hyg 45: 132−137.
56. Remme JHF (1995) The African Programme for Onchocerciasis Control: Preparing to launch. Parasitol Today 11: 403–406.
57. Amazigo UV, Obono OM, Dadzie KY, Remme J, Jiya J, et al. (2002) Monitoring community-directed treatment programmes for sustainability: Lessons from the African Programme for Onchocerciasis Control (APOC). Ann Trop Med Parasitol 96 (Suppl 1): S75−S92.
58. Molyneux DH (2005) Onchocerciasis control and elimination: Coming of age in resource-constrained health systems. Trends Parasitol 21: 525−529.
59. Gardon J, Gardon-Wendel N, Demanga-Ngangue, Kamgno J, Chippaux JP, et al. (1997) Serious reactions after mass treatment of onchocerciasis with ivermectin in an area endemic for Loa loa infection. Lancet 350: 18–22.
60. Thomson MC, Obsomer V, Kamgno J, Gardon J, Wanji S, et al. (2004) Mapping the distribution of Loa loa in Cameroon in support of the African Programme for Onchocerciasis Control. Filaria J 3: 7. 
61. African Programme for Onchocerciasis Control [APOC] (2005) External evaluation report. Ouagadougou (Burkina Faso): APOC.
62. Cook JA, Steel C, Ottesen EA (2001) Towards a vaccine for onchocerciasis. Trends Parasitol 17: 555−558.
63. Cotreau MM, Warren S, Ryan JL, Fleckenstein L, Vanapalli SR, et al. (2003) The antiparasitic moxidectin: Safety, tolerability, and pharmacokinetics in humans. J Clin Pharmacol 43: 1108–1115.
64. Trees AJ, Graham SP, Renz A, Bianco AE, Tanya V (2000) Onchocerca ochengi infections in cattle as a model for human onchocerciasis: Recent developments. Parasitology 120 (Suppl): S133–S142.
65. Gilbert J, Nfon CK, Makepeace BL, Njongmeta LM, Hastings IM, et al. (2005) Antibiotic chemotherapy of onchocerciasis: In a bovine model, killing of adult parasites requires a sustained depletion of endosymbiotic bacteria (Wolbachia species). J Infect Dis 192: 1483–1493.
66. Hoerauf A, Büttner DW, Adjei O, Pearlman E (2003) Onchocerciasis. BMJ 326: 207–210.
67. Taylor MJ, Hoerauf A (2001) A new approach to the treatment of filariasis. Curr Opin Infect Dis 14: 727–731.
68. Awadzi K, Boakye DA, Edwards G, Opoku NO, Attah SK, et al. (2004) An investigation of persistent microfilaridermias despite multiple treatments with ivermectin, in two onchocerciasis-endemic foci in Ghana. Ann Trop Med Parasitol 98: 231–249.
69. Eng JK, Prichard RK (2005) A comparison of genetic polymorphism in populations of Onchocerca volvulus from untreated- and ivermectin-treated patients. Mol Biochem Parasitol 142: 193–202.
70. Churcher TS, Ferguson NM, Basáñez M-G (2005) Density dependence and overdispersion in the transmission of helminth parasites. Parasitology 131: 121–132.
71. Duerr HP, Dietz K, Eichner M (2005) Determinants of the eradicability of filarial infections: A conceptual approach. Trends Parasitol 21: 88−96.
72. Habbema JD, Alley ES, Plaisier AP, van Oortmarssen GJ, Remme JH (1992) Epidemiological modelling for onchocerciasis control. Parasitol Today 8: 99–103.
73. Basáñez M-G, Ricárdez-Esquinca J (2001) Models for the population biology and control of human onchocerciasis. Trends Parasitol 17: 430–438.
74. Dadzie Y, Neira M, Hopkins D (2003) Final report of the Conference on the Eradicability of Onchocerciasis. Filaria J 2: 2.
75. African Programme for Onchocerciasis Control (2004) Year 2004 Progress Report of WHO/APOC. Abrufbar unter: http://www.apoc.bf/en/download.htm. Zugegriffen am 12. Januar 2006.
76. World Health Organization/African Programme for Onchocerciasis Control (2002) Onchocerciasis control in special intervention zones including Sierra Leone in the OCP area. Joint Programme Committee Report, September 2002. Ouagadougou (Burkina Faso): World Health Organization/African Programme for Onchocerciasis Control.

Abbildungen
Abb. 1. Lebenszyklus von O. volvulus
(Illustration: Giovanni Maki, adaptiert von einer Grafik des CDC auf http://www.dpd.cdc.gov/dpdx/HTML/Filariasis.htm)
DOI: 10.1371/journal.pmed.0030371.g001
Abb. 2. Verbreitung der Onchozerkose mit gegenwärtigem Status der Onchozerkosekontrolle 
In roten Gebieten findet Ivermectin-Behandlung statt. Gelbe Gebiete bedürfen weiterer epidemiologischer Untersuchungen. Grüne Gebiete sind vom Onchocerciasis Control Programme in Westafrika abgedeckt. Lila markiert frühere OCP-Gebiete, in denen Ivermectin-Behandlung und Vektorkontrollmaßnahmen durchgeführt werden. Karte abgeändert nach [53,75,76].
DOI: 10.1371/journal.pmed.0030371.g002
Abb. 3. Blindheitsinzidenz und Exzessmortalitätsrate (nach Geschlecht) aufgetragen über O. volvulus-Mikrofilarienlast. 
Arithmetisches Mittel der Mikrofilarienzahlen von zwei Skin-Snips (durchgeführt mit einer 2 mm-Holth-Hornhautstanze) von rechtem und linkem Beckenkamm. (A) Blindheit; (B) Exzessmortalitätsrate. Fehlerbalken: 95%-Konfidenzintervalle [10,22].
DOI: 10.1371/journal.pmed.0030371.g003
