# Supplementary material for: River Blindness: A Success Story under Threat?
Source: PLoS Med. 2006 Sep 26;3(9):e371. doi: 10.1371/journal.pmed.0030371 (PMC1576321; doi:10.1371/journal.pmed.0030371)
Supplement: Text S2 — (122 KB DOC). [file pmed.0030371.sd002.doc]

**Maladies Négligées**

**Cécité des Rivières: Une Réussite Menacée ?**

María-Gloria Basáñez^*^, Sébastien D. S. Pion, Thomas S. Churcher, Lutz P. Breitling, Mark P. Little, Michel Boussinesq

**Funding:** The authors received no specific funding for this article.

**Competing Interests:** The authors have declared that no competing interests exist.

**Citation:** Basáñez M-G, Pion SDS, Churcher TS, Breitling LP, Little MP, et al. (2006) River blindness: A success story under threat? PLoS Med 3(9): e371. DOI: 10.1371/journal.pmed.0030371

**DOI:** 10.1371/journal.pmed.0030371

**Copyright:** © 2006 Basáñez et al. This is an open-access article distributed under the terms of the Creative Commons Attribution License, which permits unrestricted use, distribution, and reproduction in any medium, provided the original author and source are credited.

**Abbreviations:** APOC, African Programme for Onchocerciasis Control – Programme Africain de Lutte contre l’Onchocercose; TIDC, Traitement par Ivermectine sous Directives Communautaires; OCP, Onchocerciasis Control Programme in West Africa – Programme de Lutte contre l’Onchocercose en Afrique de l’Ouest; OEPA, Onchocerciasis Elimination Program for the Americas – Programme d’Elimination de l’Onchocercose dans les Amériques; s.l., sensu lato (désigne un complexe d’espèces jumelles de simulies)

María-Gloria Basáñez, Sébastien D. S. Pion, and Thomas S. Churcher sont, respectivement, Senior Lecturer, post-doctoral research associate, et doctoral student au Department of Infectious Disease Epidemiology, et Mark P. Little est Reader au Department of Epidemiology and Public Health, Imperial College of Science, Technology and Medicine, London, United Kingdom. Lutz P. Breitling est doctoral student au Research Institute for Integrative and Comparative Biology, University of Leeds, United Kingdom. Michel Boussinesq est Directeur de Recherche à l’ Unité de recherche 024, Institut de Recherche pour le Développement, Paris, France.

* Adresse pour correspondance. E-mail: m.basanez@imperial.ac.uk

“Les réalisations de ce programme nous conduisent, nous tous, dans le domaine de la santé publique, à faire de grands rêves. Elles montrent que nous pouvons atteindre des objectifs “impossibles” et alléger le fardeau de millions de personnes, parmi les plus pauvres de la planète …” Tels furent les mots de conclusions de Gro Harlem Brundtland, alors Directrice Générale de l’Organisation mondiale de la santé, lors de la cérémonie de clôture du Programme de Lutte contre l’Onchocercose en Afrique de l’Ouest (OCP), en décembre 2002 [1]. Le succès de l’OCP est si indéniable et exemplaire, avec 600.000 cas de cécité évités, la naissance de 18 millions d’enfants dans des régions libérées du risque de cécité, et 25 millions d’hectares de terres pouvant être occupées sans risque, que la cécité des rivières est actuellement considérée comme une maladie du passé. Néanmoins, cette perception fait abstraction du fait que l’aire de l’OCP ne couvrait, au mieux, que 1,2 millions de km², que le programme ne protégeait que 30 millions de personnes vivant dans 11 pays, et qu’ailleurs en Afrique 100 millions de personnes vivent dans des zones où la transmission de l’onchocercose se poursuit. Après 28 ans de lutte, OCP nous a permis de gagner une bataille, mais une tâche beaucoup plus difficile reste à accomplir avant que l’on puisse crier victoire face à la cécité des rivières [2].

**Etiologie et Répartition**

L’onchocercose humaine est causée par un nématode parasite : la filaire *Onchocerca volvulus*. Les vers adultes (ou macrofilaires) vivent dans des nodules sous-cutanés ou profonds où les femelles, après insémination, produisent, pendant 10 ans en moyenne, les millions de microfilaires responsables des signes cliniques de l’infection. Ingérées, lors d’un repas de sang, par des vecteurs appartenant au genre *Simulium*, ou simulies, les microfilaires se développent chez ces dernières jusqu’au stade de larves infectantes (L3) qui pourront ensuite être retransmises à un autre individu (Figure 1). De nombreuses espèces de simulies peuvent, à des degrés divers, transmettre *O. volvulus* [3] et leurs rôles respectifs, en tant que vecteurs, influencent les diverses modalités de transmission du parasite au sein de son aire de répartition. En Afrique, plus de 95% des cas d’onchocercose sont dus à la transmission du parasite par des vecteurs appartenant au complexe d’espèces *Simulium damnosum* sensu lato (s.l.), qui comprend environ 60 cytotypes [3,4]. En Amérique latine, *S. ochraceum* s.l., *S. exiguum* s.l., *S. metallicum* s.l., et *S. guianense* s.l. sont les principaux vecteurs, respectivement, au Mexique et au Guatemala (environ 360.000 personnes à risque), en Colombie et en Equateur (24.600), au nord du Venezuela (104.500) et au sud du Venezuela et au Brésil (20.000) [5,6].

*O. volvulus* est endémique dans 27 pays d’Afrique subsaharienne ainsi qu’au Yémen [7], et a été importé lors de la traite des esclaves dans six pays d’Amérique latine. Il y a une dizaine d’années, on estimait que le nombre total de personnes infectées était de 18 millions [7], dont 99% vivant en Afrique. Depuis quelques années, l’utilisation de la méthode de cartographie épidémiologique rapide de l’onchocercose (*rapid epidemiological mapping of onchocerciasis*, REMO) a permis de préciser l’aire d’extension réelle de la maladie. Cette méthode consiste à sélectionner, en utilisant des critères précis, un certain nombre de villages dans chaque bassin hydrographique et évaluer le niveau d’endémie dans ces villages en mesurant la prévalence des nodules onchocerquiens sur un échantillon d’adultes [8]. En 2005, plus de 22.000 villages avaient été enquêtés de cette manière en Afrique, en dehors de l’aire d’OCP, et ceci a permis d’identifier de nombreux nouveaux foyers (Figure 2). La population vivant dans ces foyers, ainsi que celle procédant de l’accroissement démographique, dépassent certainement le nombre d’infections évitées grâce à l’OCP (on estime qu’environ 3 millions de personnes étaient infectées dans l’aire du programme [7]). Actuellement, on considère que 37 millions d’individus sont porteurs d’*O. volvulus* et que la population à risque s’élève, en Afrique, à 90 millions de personnes [9].

**Manifestations Cliniques et Pathogenèse**

L’onchocercose est plus connue sous le nom de “cécité des rivières” et cette appellation tient au fait que l’on observe, dans les villages proches des cours d’eau à courant rapide où les vecteurs se reproduisent, des taux de cécité particulièrement élevés. Il a été estimé que le nombre de sujets souffrant d’une atteinte sévère de la fonction visuelle (y compris une réduction du champ visuel) pouvait atteindre 500.000 et que par ailleurs 270.000 personnes étaient aveugles du fait de l’onchocercose [7] ; mais ces chiffres, là encore, sous-estiment certainement l’ampleur du problème. Les lésions oculaires peuvent concerner tous les tissus de l’oeil, aussi bien au niveau du segment antérieur (par exemple, les kératites ponctuées et sclérosantes) que du segment postérieur (par exemple l’atrophie optique). Le suivi d’une cohorte de sujets mise en place dans le cadre de l’OCP a permis de montrer récemment que l’incidence de la cécité est associée aux charges microfilariennes que les individus présentaient dans le passé [10]; cette observation confirme qu’une exposition continue au parasite conduit à une aggravation des atteintes oculaires onchocerquiennes (Figure 3A). Traditionnellement, on considérait que les lésions du segment antérieur étaient dues à une cascade de réactions inflammatoires induites par des antigènes filariens [11]. Selon une nouvelle hypothèse, les phénomènes pro-inflammatoires conduisant à l’opacification de la cornée seraient provoqués non seulement par le parasite lui-même, mais aussi par les bactéries endosymbiotes du genre *Wolbachia*, dont l’existence a été récemment découverte, et qui seraient libérées lors de la mort des microfilaires [12,13]. En revanche, les lésions rétiniennes, qui peuvent continuer à progresser malgré l’élimination des parasites après traitement, résulteraient de processus auto-immuns dus à des réactions croisées entre l’antigène Ov39 d’*O. volvulus* et l’antigène rétinien hr44 [14].

L’onchocercose provoque également un prurit pénible et diverses manifestations cutanées allant des lésions réactionnelles précoces – onchodermatite papulaire aiguë, onchodermatite papulaire chronique et onchodermatite lichénifiée – à des atteintes avancées telles que la dépigmentation ou l’atrophie cutanée [15]. L’onchodermatite lichénifiée ne touche parfois qu’un seul membre et est alors appelée “sowda.” La plupart des patients vivant en zone d’endémie, y compris ceux dont les charges microfilariennes dermiques sont élevées, ne présentent que des atteintes sub-cliniques ou intermittentes correspondant à une onchodermatite papulaire aiguë ; ces lésions sont associées à une faible réaction cellulaire contre les microfilaires vivantes et le tableau clinique est appelé “onchocercose généralisée.” Les lésions correspondent à un infiltrat constitué de macrophages, d’éosinophiles et de neutrophiles qui s’accumulent autour des microfilaires mortes ou en voie de destruction [16]. Comme dans la cornée, l’inflammation semble être largement induite par les bactéries symbiotiques (*Wolbachia*) libérées par les parasites [13]. Dans l’onchocercose généralisée, les réactions faisant intervenir les lymphocytes auxiliaires (*T helper cell*) de types 1 et 2 sont inhibées par un troisième type de cellules: les lymphocytes auxiliaires de type 3, ou cellules T régulatrices de type 1 [17]. Les cellules T régulatrices de type 1, spécifiques d’un antigène, produisent de grandes quantités d’interleukine 10 qui entraînent une régulation négative (*downregulation*) du système immunitaire ; celle-ci limite l’apparition des altérations liées aux phénomènes immunologiques et facilite aussi la survie du parasite [13]. A l’inverse, les patients présentant des lésions cutanées sévères ou hyper-réactives (onchodermatite lichénifiée ou sowda) ont souvent de faibles charges microfilariennes. Leurs lésions sont dues à des cycles répétés d’inflammation, avec infiltration d’éosinophiles et de macrophages, et destruction des microfilaires vivantes ou mortes [18]. Ces différents types de réponses immunitaires vis-à-vis du parasite, et les tableaux cliniques correspondants, peuvent être influencés par des facteurs génétiques de l’hôte [19].

L’onchocercose est aussi une maladie systémique, associée à des douleurs musculosquelettiques, à une réduction de l’indice de masse corporelle et à une baisse des capacités de travail. Ceci pourrait être dû au fait que les microfilaires peuvent envahir de nombreux tissus et organes, et qu’on peut les retrouver dans le sang et les urines [5]. Les infections associées à de fortes charges microfilariennes pourraient également être à l’origine de certains cas d’épilepsie [20] et du nanisme infantile connu sous le nom de syndrome de Nakalanga [21]. Il a également été montré récemment qu’il existait une relation directe entre intensité de la charge microfilarienne et mortalité des sujets [22] (Figure 3B).

**Epidémiologie**

Contrairement à ce que l’on observe pour les nématodoses intestinales et les schistosomiases, où les charges parasitaires présentent habituellement un pic chez les sujets jeunes, l’évolution avec l’âge du niveau d’infection par *O. volvulus* peut varier largement entre les foyers (les charges microfilariennes peuvent augmenter, décroître ou rester stable avec l’âge), et entre les deux sexes. Plusieurs raisons ont été avancées pour expliquer cette variabilité: différences de niveaux d’exposition au parasite selon l’âge et le sexe, facteurs endocriniens, ou immuno-suppression induite par le parasite [23,24]. Ces différences ont des implications sur la dynamique de population d’*O. volvulus* et sur l’élaboration des stratégies de lutte.

Les activités de l’OCP ont débuté en zone de savane, dans 11 pays d’Afrique de l’ouest. Ce choix était basé sur le fait que, dans ces pays, il existe deux souches parasitaires: une souche de savane, cécitante et transmise par des membres savanicoles de *S. damnosum* s.l., et une souche de forêt non cécitante, transmise par des vecteurs de forêt. Des expériences de transmission croisée avaient montré que les différents vecteurs étaient adaptés à certaines conditions locales et qu’il existait un certain degré d’incompatibilité entre les vecteurs et les parasites hétérologues. Ces observations laissaient penser qu’il existait plusieurs complexes *O. volvulus–S. damnosum* et qu’à chacun d’entre eux correspondait un type d’onchocercose, plus ou moins cécitante, couvrant une aire géographique différente de l’autre [25]. L’application de méthodes de biologie moléculaire a permis de confirmer l’existence de souches parasitaires de savane et de forêt auxquelles correspondaient, respectivement, une onchocercose oculaire sévère ou bénigne [26]. En zone de savane d’Afrique de l’ouest, la prévalence de la cécité est positivement corrélée avec l’intensité de l’infection dans la communauté. Une telle relation est rarement observée dans les zones forestières d’Afrique de l’ouest [27]. La répartition respective des deux formes d’onchocercose, à fort et relativement faible impact visuel, n’est cependant pas strictement déterminée par les limites entre savane et forêt. Des prévalences élevées de cécité peuvent ainsi être observées dans des zones de forêt et de mosaïque savane-forêt [28]. De plus, des souches d’*O. volvulus* non retrouvées en Afrique de l’ouest ont été décrites dans certains foyers. Enfin, on trouve dans d’autres régions des parasites génétiquement indifférenciables de la souche de savane d’Afrique de l’ouest mais qui ne semblent pas provoquer de cécité [29,30]. La différence de pathogénicité des diverses souches parasitaires pourrait être liée à la quantité de *Wolbachia* présente chez les parasites [31].

**Poids de la Maladie et Conséquences Socioéconomiques**

Le poids réel de l’onchocercose a été largement sous-estimé. La surmortalité des aveugles par rapport à la population générale peut être considérable, notamment chez les hommes [32,33]. Même chez des sujets non aveugles, de fortes charges microfilariennes peuvent avoir un effet négatif sur l’espérance de vie [22]. Plusieurs mécanismes pourraient être à l’origine de cette surmortalité: l’immuno-suppression, induite par le parasite, vis-à-vis d’antigènes spécifiques et non spécifiques [34], une diminution des capacités à contrôler une infection concomitante ou à répondre correctement à certaines vaccinations [35], ou certaines manifestations, telles que l’épilepsie, peut-être associées aux fortes charges parasitaires [20]. Par ailleurs, on sait bien que les lésions d’onchodermatite et l’épilepsie entraînent une stigmatisation sociale [36]. On considère que l’onchocercose est responsable de la perte annuelle d’un million d’années de vie ajustées sur l’incapacité (*disability-adjusted life-years*) – années de vie en bonne santé perdues du fait de la mortalité et de l’incapacité, dont plus de la moitié du fait des manifestations cutanées [37]). La maladie réduit de manière importante les capacités des sujets à acquérir des revenus [38], entraîne des dépenses de santé significatives et, surtout, a un impact socioéconomique immense sur les populations touchées, notamment sur leurs capacités à exploiter leurs terres [39]. Bien que l’onchocercose ne soit pas la seule cause de la dépopulation des vallées fertiles d’Afrique de l’ouest, la maladie a certainement entravé la recolonisation de ces terres arables par les populations [40]. Les bénéfices accumulés grâce aux programmes de lutte contre l’onchocercose doivent être mesurés non seulement à partir du nombre de cas de cécité évités et du rapport coût-efficacité des traitements [41,42], mais aussi en tenant compte du nombre de décès évités.

**Stratégies de Lutte contre l’Onchocercose**

La lutte contre l’onchocercose repose essentiellement sur l’élimination des vecteurs et sur l’utilisation de médicaments efficaces contre le parasite. La première stratégie est dirigée contre les stades aquatiques des simulies et la seconde, contre les microfilaires. Jusqu’à présent, il n’existe pas de médicament macrofilaricide efficace qui puisse être utilisé sans risque en traitement de masse. Les premières activités de l’OCP ont consisté à mettre en place des épandages hebdomadaires de larvicides au niveau des gîtes de reproduction des simulies, avec pour objectif d’interrompre la transmission du parasite au niveau de l’aire initiale du programme. Une fois ce résultat atteint, il s’est agi d’éliminer le parasite dans la population. Pour ce faire il était nécessaire de tarir les sources de nouveaux vecteurs et ce pendant aussi longtemps que des microfilaires seraient présentes dans la peau des personnes vivant dans l’aire considérée. Compte tenu de la longévité à la fois des vers adultes et des microfilaires, on estimait que la période nécessaire serait d’au moins 14 ans [43]. Dans certaines régions de l’aire d’OCP, il a été prouvé que les enfants nés après la mise en place de la lutte antivectorielle sont restés non infectés [44]. En 1987, les laboratoires Merck & Co. prirent la décision sans précédent de donner l’ivermectine (Mectizan^TM^), un médicament microfilaricide et sans danger, et ce pour aussi longtemps que nécessaire, afin d’éliminer l’onchocercose en tant que problème de santé publique. Suite à cet engagement, des distributions régulières d’ivermectine par des équipes mobiles furent organisées dans l’aire d’OCP, soit en complément de la lutte antivectorielle, soit comme seule intervention [45]. L’ivermectine, administrée à la dose de 150 microgramme par kilo de poids, possède une activité microfilaricide très marquée et empêche pendant plusieurs mois la production de nouvelles microfilaires par les vers femelles. Les traitements de masse par ivermectine de toute la population âgée de cinq ans ou plus (à l’exception des femmes enceintes ou allaitant un enfant de moins d’une semaine), répétés une ou deux fois par an, entraînent une réduction de la morbidité et de l’incapacité [46,47] et une baisse de l’intensité de transmission du parasite [48,49]. Dans les foyers présentant des niveaux d’endémie très élevés, on considère que les traitements annuels ne permettront pas d’éliminer localement la population parasitaire [50], à moins que des couvertures thérapeutiques très élevées (plus de 80% de la population totale) soient maintenues pendant au moins 25 ans, et ce sans réduction de l’efficacité du traitement [51].

En Amérique latine, des activités limitées de lutte antivectorielle ont été menées avec un certain succès au Guatemala, contre le vecteur local *S. ochraceum* s.l. [52]; toutefois, cette stratégie n’a pas été jugée utilisable ailleurs. Le Programme d’Elimination de l’Onchocercose dans les Amériques (OEPA), lancé en 1993, est un partenariat régional dont l’objectif est d’éliminer l’ensemble de la morbidité d’origine onchocerquienne (et d’éliminer la transmission quand cela est possible) dans les foyers des six pays endémiques d’Amérique latine [52]. Considérant que des traitements administrés tous les six mois auraient un plus grand impact sur la transmission du parasite [54] et sur la fertilité des vers femelles [55], l’OEPA utilise actuellement une stratégie de distributions de masse biannuelles.

Le Programme Africain de Lutte contre l’Onchocercose (APOC) a été lancé en 1995 afin de couvrir les 19 pays africains situés en dehors de l’aire d’OCP [56] (trois d’entre eux, le Kenya, le Rwanda et le Mozambique se sont depuis révélés être non endémiques). Depuis l’origine, la stratégie de l’APOC est basée sur les distributions annuelles d’ivermectine. Les couvertures géographiques (pourcentage de villages traités dans une région) et thérapeutiques (pourcentage de la population traitée dans un village) atteintes quand les traitements étaient effectués par des équipes mobiles n’étaient pas satisfaisantes. Par ailleurs, cette stratégie de traitement ne permettait probablement pas d’assurer une durabilité des distributions. C’est pourquoi l’APOC a développé, avec grand succès, une stratégie de traitement par ivermectine sous directives communautaires (TIDC). Dans ce cadre, les communautés elles-mêmes désignent des distributeurs locaux qui sont responsables du traitement [57]. Fin 2005, 400 millions de traitements au total avaient été fournis par le Programme de donation du Mectizan, et l’on estime que 40 millions de personnes, vivant dans 90.000 villages africains, avaient été traités par 300.000 distributeurs communautaires dans le cadre des projets soutenus par l’APOC.

Le coût moyen par personne traitée, en tenant compte du temps passé par les distributeurs, est de 0,74 US$. Ce montant fait du TIDC une activité très rentable en terme de rapport coût-efficacité [9]. Par ailleurs, le coût par personne traitée dans le cadre de l’APOC (hors coût du Mectizan) est presque 8,5 fois inférieur au montant qui était nécessaire pour protéger une personne, grâce à la lutte antivectorielle, dans le cadre de l’OCP [42]. De plus, la stratégie du TIDC a permis de conférer aux communautés une responsabilité telle que le TIDC est actuellement utilisé comme une plate-forme permettant d’intégrer d’autres interventions à base communautaire, notamment la distribution d’autres médicaments (par exemple : supplémentation en vitamine A et distribution d’albendazole pour lutter contre la filariose lymphatique). Cette intégration du TIDC avec d’autres programmes de lutte pourrait faciliter le maintien de couvertures thérapeutiques élevées, alors même que la morbidité onchocerquienne décroît dans la population [58]. Toutefois, en dépit de ses résultats impressionnants en terme de couverture, et malgré les perspectives prometteuses d’interventions sous directives communautaires combinées, l’APOC doit surmonter certaines difficultés sérieuses avant d’atteindre ses objectifs ultimes : durabilité sur le long terme et maintien d’un impact permanent des traitements.

Dans les régions (principalement d’Afrique centrale) où l’onchocercose et la loase (causée par la filaire *Loa loa*) sont co-endémiques, le traitement de l’onchocercose par ivermectine peut entraîner, chez les sujets présentant une microfilarémie à *L. loa* élevée, des effets secondaires sévères, y compris une encéphalopathie fatale [59]. Cette situation a considérablement freiné l’expansion de l’APOC. Des modèles géostatistiques sont développés pour délimiter les zones où la loase est hyperendémique [60], et divers protocoles vont être testés en vue d’identifier un traitement permettant de réduire la microfilarémie à *L. loa* avant la mise en place des traitements par ivermectine.

Des études visant à évaluer la durabilité des projets parrainés par l’APOC ont également montré que les communautés ne soutiennent pas toujours de manière adéquate leurs distributeurs. L’engagement de ces derniers se maintient souvent du fait de leur implication dans d’autres activités plus “lucratives”, telles que les campagnes de vaccination. Faute de moyens, les supervisions au niveau des communautés et des structures de santé est parfois difficile, et de nombreux obstacles doivent encore être surmontés avant que le TIDC puisse être intégré avec succès à d’autres activités sanitaires [61].

Ces problèmes font que l’on se demande jusqu’à quand le programme doit se poursuivre. Lors de son lancement, il était prévu que la durée de l’APOC serait de 12 ans (de 1995 à fin 2007). Depuis lors, une période de désengagement de deux ans a été ajoutée et le soutien financier des pays donateurs est assuré jusqu’à 2010. Actuellement, aucune décision n’a été prise quant à une nouvelle prolongation mais, étant données les caractéristiques du parasite et de ses vecteurs, il est probable que les activités de l’APOC devraient être maintenues pendant au moins 20 ans si l’on veut obtenir un impact significatif et durable [42].

**Nécessité d’Autres Médicaments Efficaces contre *O. volvulus***

Le fait que la lutte contre l’onchocercose repose de plus en plus sur l’ivermectine seule et l’absence d’avancée réelle dans le développement d’un vaccin [62] ont stimulé la recherche d’autres composés actifs contre *O. volvulus*. Ainsi, la moxidectine est un microfilaricide extrêmement efficace dont la demi-vie chez l’homme est plus longue que celle de l’ivermectine [63]; en conséquence, ce médicament pourrait inhiber pendant plus longtemps que l’ivermectine la fertilité des vers adultes [63]. La structure chimique de la moxidectine est similaire à celle de l’ivermectine et, chez des modèles animaux, elle ne semble pas avoir de réel effet macrofilaricide [64].

L’élimination prolongée des *Wolbachia* entraînant une baisse de la reproduction et de la longévité des vers [65], l’utilisation d’antibiotiques pourrait constituer une nouvelle approche thérapeutique. Des traitements à la dose de 100 milligrammes par jour pendant six semaines (ou de 200 milligrammes par jour pendant quatre semaines) entraînent une interruption de l’embryogenèse qui dure au moins 18 mois [66]. Cependant, la durée du traitement, les diverses contre-indications aux antibiotiques, et le risque d’induire des résistances chez d’autres agents pathogènes font qu’il est difficile d’envisager cette option dans le cadre de traitements de masse. Les recherches sur l’activité d’autres antibiotiques et sur la durée minimum de traitement nécessaire pour éliminer de façon permanente les *Wolbachia* pourraient permettre de surmonter certains de ces obstacles [67]. Mais les médicaments actifs sur les *Wolbachia* pourraient être utilisés pour traiter sélectivement les individus présentant toujours des microfilaires à la fin des traitements de masse par ivermectine et de “nettoyer” ainsi les zones où l’élimination du parasite est jugée possible.

On peut s’attendre à ce que l’extension des divers programmes reposant sur l’utilisation de l’ivermectine (dans les pays de l’ex-OCP et dans ceux couverts par l’APOC et l’OEPA) imposera une pression de sélection sur le génome du parasite. Bien qu’à ce jour aucun cas confirmé de résistance à l’ivermectine n’ait été identifié, un phénotype de réponse sub-optimale au médicament a été décrit dans des localités du Ghana chez des sujets ayant reçu plus de neuf traitements [68]. Ce phénomène semble être dû non pas à une baisse de l’effet microfilaricide du médicament, mais à une reprise plus précoce après traitement des capacités de reproduction des femelles adultes. Une étude comparant le génotype de parasites collectés soit chez des sujets n’ayant jamais été traités par ivermectine, soit chez des individus ayant reçu au moins six traitements annuels a permis de mettre en évidence une sélection génétique au niveau de loci polymorphes associés à une résistance à l’ivermectine chez des nématodes animaux [69]. Toutefois, les études permettant de relier le phénotype de réponse au médicament et le génotype du parasite au fur et à mesure des traitements successifs restent à effectuer. Des modèles mathématiques pourraient aider à mieux comprendre les processus qui, dans le cadre de la dynamique de population du parasite, pourraient influencer les modalités de recrudescence de l’infection [70,71] et la diffusion des allèles favorisés par la sélection induite par l’ivermectine.

**Apport de la Modélisation dans le Cadre de la Lutte contre l’Onchocercose**

Dans l’histoire de la lutte contre les parasitoses, l’onchocercose est exemplaire dans la mesure où les stratégies d’intervention ont été guidées à tous les stades par des modèles de simulation informatique. Le modèle ONCHOSIM, en particulier, a été développé sous les auspices de l’OCP pour modéliser l’onchocercose en zone de savane ouest-africaine [72]. D’autres modèles ont été élaborés pour décrire la transmission et guider la lutte dans les zones de forêt et dans les foyers latino-américains [73]. Les réponses à la question-clé de savoir combien de temps les traitements antifilariens doivent être poursuivis dépendent des objectifs poursuivis et de l’épidémiologie particulière de l’onchocercose dans les foyers considérés. Si l’objectif est d’éliminer l’onchocercose en tant que problème de santé publique, les traitements annuels par ivermectine (tels qu’organisés par l’APOC) constitueront une stratégie efficace dès lors que l’on parvient à abaisser l’intensité de l’infection à des valeurs inférieures à cinq ou dix microfilaires par biopsie ; toutefois, cette stratégie ne permettra probablement pas d’interrompre la transmission d’*O. volvulus* en Afrique [74]. L’intensité et la saisonalité de la transmission, le complexe *Onchocerca–Simulium* en cause, la répartition des parasites chez les hôtes, les processus de densité-dépendance agissant aux différents stades du cycle parasitaire, ainsi que les interactions entre ces différents facteurs et les modalités et la couverture des interventions constituent autant de facteurs qui déterminent la stabilité du système hôte-parasite et notre capacité (ou notre incapacité) à “pousser” *O. volvulus* au dessous d’un éventuel seuil critique de transmission [70,71,73].

**Conclusion**

Le désintérêt porté à la lutte contre l’onchocercose peut se manifester de diverses manières. Un engagement financier et politique est nécessaire non seulement pour soutenir les programmes de lutte mais aussi pour financer la recherche permettant de développer les outils requis pour éliminer le parasite. Du fait du succès spectaculaire de l’OCP, l’onchocercose bénéficie actuellement tout d’un très mauvais classement dans la liste des priorités de la recherche en santé. Ceci est fort dommageable car nous sommes à un moment où il est essentiel de consolider les acquis des activités passées et où le succès à long terme de l’APOC et de l’OEPA reste à démontrer. La priorité doit être donnée au développement de meilleurs outils de diagnostic (la technique parasitologique de détection des microfilaires dermiques perdra sa sensibilité au fur et à mesure que la lutte progressera, et les tests de détection des antigènes parasitaires ne donnent pas de résultats tangibles), à la recherche de médicaments macrofilaricides, à la détection précoce d’une éventuelle baisse de l’efficacité des médicaments (et à la sélection génétique associée chez le parasite), et à la meilleure compréhension de l’impact des traitements de masse sur la dynamique de population d’*O. volvulus*. Les perspectives actuelles, selon lesquelles les distributions d’ivermectine devront peut-être être poursuivies indéfiniment, font courir le risque d’une apparition d’une résistance aux médicaments ; elles pourraient également susciter une certaine lassitude de l’opinion publique et des donateurs.

**Remerciements**

Les idées discutées ici sont le produit de travaux pour lesquels les auteurs ont bénéficié de divers soutiens financiers au cours des années passées. Nous remercions ainsi le Wellcome Trust (MGB), le Medical Research Council UK (MGB et TSC), la Fondation pour la Recherche Médicale et la Fondation Singer-Polignac, France (SDSP), la River Blindness Foundation et le Programme spécial UNICEF/PNUD/Banque mondiale/OMS pour la recherche et la formation concernant les maladies tropicales (MB).

**Références**

1. http://www.who.int/dg/speeches/2002/Ouagadougou/en

2. Hopkins AD (2005) Ivermectin and onchocerciasis: Is it all solved? Eye 19: 1057–1066.

3. Crosskey RW (1990) The natural history of blackflies. Chichester: John Wiley & Sons. 711 p.

4. Crosskey RW, Howard TM (2004) A revised taxonomic and geographical inventory of world blackflies (Diptera : Simuliidae). London: The Natural History Museum. Available: http://www.nhm.ac.uk/research-curation/projects/blackflies. Accessed 24 April 2006.

5. Bradley JE, Whitworth J, Basáñez M-G (2005) Onchocerciasis. In: Cox FEG, Wakelin D, Gillespie SH, Despommier DD. Parasitology, Topley and Wilson’s microbiology and microbial infections. 10th edition. London: Hodder Arnold. pp. 781–801.

6. World Health Organization (2005) Onchocerciasis (river blindness). Weekly epidemiological record 80: 257–260. Available: http://www.who.int/wer/2005/wer8030.pdf. Accessed 26 July 2006.

7. World Health Organization (1995) Onchocerciasis and its control. Report of a WHO expert committee on onchocerciasis control. WHO Technical Report Series, number 852. Geneva: World Health Organization. 110 p.

8. Ngoumou P, Walsh JF, Macé JM (1994) A rapid mapping technique for the prevalence and distribution of onchocerciasis: A Cameroon case study. Ann Trop Med Parasitol 88: 463–474.

9. African Programme for Onchocerciasis Control [APOC] (2005) Final communiqué of the 11th session of the Joint Action Forum (JAF) of APOC, Paris, France, 6–9 December 2005. Ouagadougou (Burkina Faso): APOC.

10. Little MP, Basáñez M-G, Breitling LP, Boatin BA, Alley ES (2004) Incidence of blindness during the entire duration of the Onchocerciasis Control Programme in western Africa, 1971–2002. J Infect Dis 189: 1932–1941.

11. Hall LR, Pearlman E (1999) Pathogenesis of onchocercal keratitis (river blindness). Clin Microbiol Rev 12: 445–453.

12. Saint-André A, Blackwell NM, Hall LR, Hoerauf A, Brattig NW, et al. (2002) The role of endosymbiotic *Wolbachia* bacteria in the pathogenesis of river blindness. Science 295: 1892–1895.

13. Brattig NW (2004) Pathogenesis and host responses in human onchocerciasis: Impact of *Onchocerca* filariae and *Wolbachia* endobacteria. Microbes Infect 6: 113–128.

14. McKechnie NM, Gürr W, Yamada H, Copland D, Braun G (2002) Antigenic mimicry: *Onchocerca volvulus* antigen-specific T cells and ocular inflammation. Invest Ophthalmol Vis Sci 43: 411–418.

15. Murdoch ME, Hay RJ, Mackenzie CD, Williams JF, Ghalib HW, et al. (1993) A clinical classification and grading system of the cutaneous changes in onchocerciasis. Br J Dermatol 129: 260–269.

16. Pearlman E, Garhart CA, Grand DJ, Diaconu E, Strine ER, et al. (1999) Temporal recruitment of neutrophils and eosinophils to the skin in a murine model for onchocercal dermatitis. Am J Trop Med Hyg 61: 14–18.

17. Hoerauf A, Brattig N (2002) Resistance and susceptibility in human onchocerciasis—Beyond Th1 vs. Th2. Trends Parasitol 18: 25–31.

18. Ali MM, Baraka OZ, AbdelRahman SI, Sulaiman SM, Williams JF, et al. (2003) Immune responses directed against microfilariae correlate with severity of clinical onchodermatitis and treatment history. J Infect Dis 187: 714–717.

19. Meyer CG, Gallin M, Erttmann KD, Brattig N, Schnittger L, et al. (1994) HLA-D alleles associated with generalized disease, localized disease, and putative immunity in *Onchocerca volvulus* infection. Proc Natl Acad Sci U S A 91: 7515–7519.

20. Boussinesq M, Pion SD, Demanga-Ngangue, Kamgno J (2002) Relationship between onchocerciasis and epilepsy: A matched case-control study in the Mbam Valley, Republic of Cameroon. Trans R Soc Trop Med Hyg 96: 537–541.

21. Kipp W, Burnham G, Bamuhiiga J, Leichsenring M (1996) The Nakalanga syndrome in Kabarole district, Western Uganda. Am J Trop Med Hyg 54: 80–83.

22. Little MP, Breitling LP, Basáñez M-G, Alley ES, Boatin BA (2004) Association between microfilarial load and excess mortality in human onchocerciasis: An epidemiological study. Lancet 363: 1514–1521.

23. Filipe JA, Boussinesq M, Renz A, Collins RC, Vivas-Martinez S, et al. (2005) Human infection patterns and heterogeneous exposure in river blindness. Proc Natl Acad Sci U S A 102: 15265–15270.

24. Duerr HP, Dietz K, Schulz-Key H, Büttner DW, Eichner M (2003) Density-dependent parasite establishment suggests infection-associated immunosuppression as an important mechanism for parasite density regulation in onchocerciasis. Trans R Soc Trop Med Hyg 97: 242−250.

25. Duke BO, Lewis DJ, Moore PJ (1966) *Onchocerca-Simulium* complexes. I. Transmission of forest and Sudan-savanna strains of *Onchocerca volvulus*, from Cameroon, by *Simulium damnosum* from various West African bioclimatic zones. Ann Trop Med Parasitol 60: 318–336.

26. Zimmerman PA, Dadzie KY, De Sole G, Remme J, Alley ES, et al. (1992) *Onchocerca volvulus* DNA probe classification correlates with epidemiologic patterns of blindness. J Infect Dis 165: 964–968.

27. Dadzie KY, Remme J, Rolland A, Thylefors B (1989) Ocular onchocerciasis and intensity of infection in the community. II. West African rainforest foci of the vector *Simulium yahense*. Trop Med Parasitol 40: 348–354.

28. Kayembe DL, Kasonga D L, Kayembe PK, Mwanza JC, Boussinesq M (2003) Profile of eye lesions and vision loss: A cross-sectional study in Lusambo, a forest-savanna area hyperendemic for onchocerciasis in the Democratic Republic of Congo. Trop Med Int Health 8: 83–89.

29. Fischer P, Bamuhiiga J, Kilian AH, Büttner DW (1996) Strain differentiation of *Onchocerca volvulus* from Uganda using DNA probes. Parasitology 112: 401–408.

30. Higazi TB, Katholi CR, Mahmoud BM, Baraka OZ, Mukhtar MM, et al. (2001) *Onchocerca volvulus*: Genetic diversity of parasite isolates from Sudan. Exp Parasitol 97: 24–34.

31. Higazi TB, Filiano A, Katholi CR, Dadzie Y, Remme JH, et al. (2005) *Wolbachia* endosymbiont levels in severe and mild strains of *Onchocerca volvulus*. Mol Biochem Parasitol 141: 109–112.

32. Prost A (1986) The burden of blindness in adult males in the savanna villages of West Africa exposed to onchocerciasis. Trans R Soc Trop Med Hyg 80: 525–527.

33. Pion SD, Kamgno J, Demanga-Ngangue, Boussinesq M (2002) Excess mortality associated with blindness in the onchocerciasis focus of the Mbam Valley, Cameroon. Ann Trop Med Parasitol 96: 181–189.

34. Stewart GR, Boussinesq M, Coulson T, Elson L, Nutman T, et al. (1999) Onchocerciasis modulates the immune response to mycobacterial antigens. Clin Exp Immunol 117: 517–523.

35. Cooper PJ, Espinel I, Paredes W, Guderian RH, Nutman TB (1998) Impaired tetanus-specific cellular and humoral responses following tetanus vaccination in human onchocerciasis: A probable role for interleukin-10. J Infect Dis 178: 1133–1138.

36. Vlassoff C, Weiss M, Ovuga EB, Eneanya C, Newel PT, et al. (2000) Gender and the stigma of onchocercal skin disease in Africa. Soc Sci Med 50: 1353–1368.

37. Remme JH (2004) Research for control: The onchocerciasis experience. Trop Med Int Health 9: 243–254.

38. Oladepo O, Brieger WR, Otusanya S, Kale OO, Offiong S, et al. (1997) Farm land size and onchocerciasis status of peasant farmers in south-western Nigeria. Trop Med Int Health 2: 334–340.

39. Evans TG (1995) Socioeconomic consequences of blinding onchocerciasis. Bull World Health Organ 73: 495–506.

40. Hervouët JP, Prost A (1979) Organisation de l'espace et épidémiologie de l'onchocercose. In: Maîtrise de l'espace agraire et développement en Afrique tropicale. Mémoires ORSTOM, number 89. Paris: ORSTOM. pp. 179–190.

41. Benton B (1998) Economic impact of onchocerciasis control through the African Programme for Onchocerciasis Control: An overview. Ann Trop Med Parasitol 92 (Suppl): S33–S39.

42. Waters HR, Rehwinkel JA, Burnham G (2004) Economic evaluation of Mectizan distribution. Trop Med Int Health 9 (Suppl): A16–A25.

43. Hougard JM, Alley ES, Yaméogo L, Dadzie KY, Boatin BA (2001) Eliminating onchocerciasis after 14 years of vector control: A proved strategy. J Infect Dis 184: 497−503.

44. Ba O, Karam M, Remme J, Zerbo G (1987) Place des enfants dans l'évaluation du programme de lutte contre l'onchocercose en Afrique de l'ouest [Role of children in the evaluation of the Onchocerciasis Control Program in West Africa]. Trop Med Parasitol 38: 137–142.

45. Molyneux DH (1995) Onchocerciasis control in West Africa: Current status and future of the Onchocerciasis Control Programme. Parasitol Today 11: 399–402.

46. Ejere H, Schwartz E, Wormald R (2001) Ivermectin for onchocercal eye disease (river blindness). Cochrane Database Syst Review: 2001: CD002219.

47. Tielsch JM, Beeche A (2004) Impact of ivermectin on illness and disability associated with onchocerciasis. Trop Med Int Health 9 (Suppl): A45–A56.

48. Boussinesq M, Prod'hon J, Chippaux JP (1997) *Onchocerca volvulus*: Striking decrease in transmission in the Vina valley (Cameroon) after eight annual large scale ivermectin treatments. Trans R Soc Trop Med Hyg 91: 82−86.

49. Collins RC, Gonzalez-Peralta C, Castro J, Zea-Flores G, Cupp MS, et al. (1992) Ivermectin: Reduction in prevalence and infection intensity of *Onchocerca volvulus* following biannual treatments in five Guatemalan communities. Am J Trop Med Hyg 47: 156−169.

50. Borsboom GJ, Boatin BA, Nagelkerke NJ, Agoua H, Akpoboua KL, et al. (2003) Impact of ivermectin on onchocerciasis transmission: Assessing the empirical evidence that repeated ivermectin mass treatments may lead to elimination/eradication in West Africa. Filaria J 2: 8.

51. Winnen M, Plaisier AP, Alley ES, Nagelkerke NJ, van Oortmarssen G, et al. (2002) Can ivermectin mass treatments eliminate onchocerciasis in Africa? Bull World Health Organ 80: 384–390.

52. Ochoa JO, Castro JC, Barrios VM, Juarez EL, Tada I (1997) Successful control of onchocerciasis vectors in San Vicente Pacaya, Guatemala, 1984–1989. Ann Trop Med Parasitol 91: 471−479.

53. Richards FO, Boatin B, Sauerbrey M, Sékétéli A (2004) Control of onchocerciasis today: Status and challenges. Trends Parasitol 17: 558–563.

54. Cupp EW, Ochoa JO, Collins RC, Cupp MS, Gonzalez-Peralta C, et al. (1992) The effects of repetitive community-wide ivermectin treatment on transmission of *Onchocerca volvulus* in Guatemala. Am J Trop Med Hyg 47: 170−180.

55. Duke BO, Zea-Flores G, Castro J, Cupp EW, Muñoz B (1991) Comparison of the effects of a single dose and of four six monthly doses of ivermectin on adult *Onchocerca volvulus*. Am J Trop Med Hyg 45: 132−137.

56. Remme JHF (1995) The African Programme for Onchocerciasis Control: Preparing to launch. Parasitol Today 11: 403–406.

57. Amazigo UV, Obono OM, Dadzie KY, Remme J, Jiya J, et al. (2002) Monitoring community-directed treatment programmes for sustainability: Lessons from the African Programme for Onchocerciasis Control (APOC). Ann Trop Med Parasitol 96 (Suppl 1): S75−S92.

58. Molyneux DH (2005) Onchocerciasis control and elimination: Coming of age in resource-constrained health systems. Trends Parasitol 21: 525−529.

59. Gardon J, Gardon-Wendel N, Demanga-Ngangue, Kamgno J, Chippaux JP, et al. (1997) Serious reactions after mass treatment of onchocerciasis with ivermectin in an area endemic for *Loa loa* infection. Lancet 350: 18–22.

60. Thomson MC, Obsomer V, Kamgno J, Gardon J, Wanji S, et al. (2004) Mapping the distribution of *Loa loa* in Cameroon in support of the African Programme for Onchocerciasis Control. Filaria J 3: 7.

61. African Programme for Onchocerciasis Control [APOC] (2005) External evaluation report. Ouagadougou (Burkina Faso): APOC.

62. Cook JA, Steel C, Ottesen EA (2001) Towards a vaccine for onchocerciasis. Trends Parasitol 17: 555−558.

63. Cotreau MM, Warren S, Ryan JL, Fleckenstein L, Vanapalli SR, et al. (2003) The antiparasitic moxidectin: Safety, tolerability, and pharmacokinetics in humans. J Clin Pharmacol 43: 1108–1115.

64. Trees AJ, Graham SP, Renz A, Bianco AE, Tanya V (2000) *Onchocerca ochengi* infections in cattle as a model for human onchocerciasis: Recent developments. Parasitology 120 (Suppl): S133–S142.

65. Gilbert J, Nfon CK, Makepeace BL, Njongmeta LM, Hastings IM, et al. (2005) Antibiotic chemotherapy of onchocerciasis: In a bovine model, killing of adult parasites requires a sustained depletion of endosymbiotic bacteria (*Wolbachia* species). J Infect Dis 192: 1483–1493.

66. Hoerauf A, Büttner DW, Adjei O, Pearlman E (2003) Onchocerciasis. BMJ 326: 207–210.

67. Taylor MJ, Hoerauf A (2001) A new approach to the treatment of filariasis. Curr Opin Infect Dis 14: 727–731.

68. Awadzi K, Boakye DA, Edwards G, Opoku NO, Attah SK, et al. (2004) An investigation of persistent microfilaridermias despite multiple treatments with ivermectin, in two onchocerciasis-endemic foci in Ghana. Ann Trop Med Parasitol 98: 231–249.

69. Eng JK, Prichard RK (2005) A comparison of genetic polymorphism in populations of *Onchocerca volvulus* from untreated- and ivermectin-treated patients. Mol Biochem Parasitol 142: 193–202.

70. Churcher TS, Ferguson NM, Basáñez M-G (2005) Density dependence and overdispersion in the transmission of helminth parasites. Parasitology 131: 121–132.

71. Duerr HP, Dietz K, Eichner M (2005) Determinants of the eradicability of filarial infections: A conceptual approach. Trends Parasitol 21: 88−96.

72. Habbema JD, Alley ES, Plaisier AP, van Oortmarssen GJ, Remme JH (1992) Epidemiological modelling for onchocerciasis control. Parasitol Today 8: 99–103.

73. Basáñez M-G, Ricárdez-Esquinca J (2001) Models for the population biology and control of human onchocerciasis. Trends Parasitol 17: 430–438.

74. Dadzie Y, Neira M, Hopkins D (2003) Final report of the Conference on the Eradicability of Onchocerciasis. Filaria J 2: 2.

75. African Programme for Onchocerciasis Control (2004) Year 2004 Progress Report of WHO/APOC. Available: http://www.apoc.bf/en/download.htm. Accessed 12 January 2006.

76. World Health Organization/African Programme for Onchocerciasis Control (2002) Onchocerciasis control in special intervention zones including Sierra Leone in the OCP area. Joint Programme Committee Report, September 2002. Ouagadougou (Burkina Faso): World Health Organization/African Programme for Onchocerciasis Control.

**Figure 1.** Cycle Parasitaire d’*O. volvulus*.

(Illustration: Giovanni Maki, derived from a CDC image at http://www.dpd.cdc.gov/dpdx/HTML/Filariasis.htm)

DOI: 10.1371/journal.pmed.0030371.g001

**Figure 2.** Carte de Répartition de l’Onchocercose Indiquant le Statut Actuel des Programmes de Lutte.

Les régions en rouge indiquent les zones où des traitements par ivermectine sont organisés. Les régions en jaune montrent les zones où des enquêtes épidémiologiques supplémentaires sont nécessaires. La zone en vert est celle qui était couverte par le Programme de Lutte contre l’Onchocercose en Afrique de l’Ouest (OCP). Les zones en violet correspondent aux Zones d’intervention spéciales, c’est-à-dire des zones de l’ex-OCP traitées par ivermectine et où des opérations de lutte anti-vectorielle sont organisées. Carte redessinée à partir de [53,75,76].

DOI: 10.1371/journal.pmed.0030371.g002

**Figure 3.** Taux d’Incidence de la Cécité et Taux de Surmortalité, par Sexe, en Fonction des Charges Microfilariennes à *O. volvulus*.

Moyenne arithmétique du nombre de microfilaires issues de deux biopsies cutanées prélevées aux crêtes iliaques droite et gauche en utilisant une pince à sclérectomie type Holth 2 millimètres. (A) Cécité; (B) Taux de surmortalité. Les barres indiquent les intervalles de confiance à 95 pour cent [10,22].

DOI: 10.1371/journal.pmed.0030371.g003
