# Supplementary material for: River Blindness: A Success Story under Threat?
Source: PLoS Med. 2006 Sep 26;3(9):e371. doi: 10.1371/journal.pmed.0030371 (PMC1576321; doi:10.1371/journal.pmed.0030371)
Supplement: Text S3 — (100 KB DOC). [file pmed.0030371.sd003.doc]

**Enfermedades Abandonadas**

**Ceguera de los Ríos: La historia de un éxito en peligro?**

María-Gloria Basáñez^*^, Sébastien D. S. Pion, Thomas S. Churcher, Lutz P. Breitling, Mark P. Little, Michel Boussinesq

**Financiamiento:** Los autores no recibieron financiamiento específico para la preparación de este artículo.

**Conflicto de Intereses :** Los autores han declarado que no existen conflictos de interés.

**Citación:** Basáñez M-G, Pion SDS, Churcher TS, Breitling LP, Little MP, et al. (2006) River blindness: A success story under threat? PLoS Med 3(9): e371. DOI: 10.1371/journal.pmed.0030371

**DOI:** 10.1371/journal.pmed.0030371

**Copyright:** © 2006 Basáñez et al. Este es un artículo de acceso abierto distribuido bajo los términos de la “Creative Commons Attribution License”, la cual permite el uso irrestringido, la distribución y reproducción en cualquier medio, siempre y cuando los autores y fuente originales sean acreditadas.

**Abreviaturas:** APOC, Programa Africano para el Control de la Oncocercosis; TIDC, Tratamiento con Ivermectina Dirigido por las Comunidades; OCP, Programa de Lucha contra la Oncocercosis en África Occidental ; OEPA, Programa para la Eliminación de la Oncocercosis en las Américas; s.l., sensu lato (designa un complejo de especies gemelas o especies crípticas, difícilmente distinguibles morfológicamente pero que pueden diferenciarse mediante estudios citogenéticos)

María-Gloria Basáñez, Sébastien D. S. Pion, y Thomas S. Churcher son, respectivamente, Senior Lecturer, investigador asociado de post-doctorado, y estudiante de doctorado en el Departamento de Epidemiología de Enfermedades Infecciosas, y Mark P. Little es Reader en el Departamento de Epidemiología y Salud Publica, Imperial College, Londres, Reino Unido. Lutz P. Breitling es estudiante de doctorado en el Instituto de Investigación para la Biología Integrada y Comparativa , Universidad de Leeds, Reino Unido. Michel Boussinesq es Director de Investigación en la Unidad de investigación 024, Instituto de Investigación para el Desarrollo, Paris, Francia.

* Correspondencia: m.basanez@imperial.ac.uk

“Los logros de este programa inspiran a todos aquellos que trabajamos en salud pública a soñar grandes ideales. Demuestran que podemos alcanzar metas “imposibles” y aligerar la carga de millones de las personas más pobres del planeta …” Tales fueron las palabras finales de Gro Harlem Brundtland, entonces Directora General de la Organización Mundial de la Salud, durante la ceremonia de clausura del Programa de Lucha contra la Oncocercosis en África Occidental (OCP), en diciembre de 2002 [1]. El éxito de este programa es tan innegable y ejemplar (con 600.000 casos de ceguera prevenidos, 18 millones de niños nacidos en regiones liberadas del riesgo de ceguera por oncocercosis, y 25 millones de hectáreas de tierra liberadas para su repoblamiento), que la ceguera de los ríos está actualmente considerada como una enfermedad del pasado. Esta percepción, no obstante, olvida el hecho de que el área cubierta por OCP fue a lo sumo de 1,2 millones de kilómetros cuadrados, protegiendo a 30 millones de personas en 11 países, pero dejando por fuera a 100 millones de personas en el resto del continente, quienes viven en áreas donde aún existe transmisión activa. Luego de 28 años de lucha, el éxito de OCP nos ha permitido ganar una batalla, pero una tarea mucho más difícil nos aguarda antes que podamos clamar victoria contra la ceguera de los ríos [2].

**Etiología y Distribución**

La oncocercosis humana es causada por un parásito nemátodo: la filaria *Onchocerca volvulus*. Los vermes adultos (o macrofilarias) viven dentro de nódulos subcutáneos o profundos donde las hembras, luego de ser inseminadas, producen, durante unos 10 años en promedio, millones de microfilarias responsables de los signos clínicos de la infección. Ingeridas, junto con una comida sanguínea por parte de insectos vectores del género *Simulium*, o simúlidos, las microfilarias se desarrollan dentro de éstos hasta alcanzar el estadio de larvas infectantes (L3), que pueden ser trasmitidas a otros individuos (Figura 1). Numerosas especies de simúlidos participan, en diversos grados, en la transmisión de *O. volvulus* [3], y sus papeles vectoriales relativos ejercen una influencia importante sobre los patrones epidemiológicos en áreas endémicas. En África, el complejo de especies gemelas *Simulium damnosum* sensu lato (s.l.), el cual comprende unas 60 citoformas, es responsable por más del 95% de los casos de oncocercosis a nivel mundial, [3,4]. En Latinoamérica, *S. ochraceum* s.l., *S. exiguum* s.l., *S. metallicum* s.l., y *S. guianense* s.l. son los vectores principales, respectivamente, en México y Guatemala (unas 360.000 personas a riesgo), Colombia y Ecuador (24.600), norte de Venezuela (104.500), y sur de Venezuela y Brasil (20.000) [5,6].

La infección por *O. volvulus* es endémica en 27 países de África al sur del Sahara, en un país de la península arábica (Yemen) [7], y en seis países de América Latina (donde fue introducida durante el tráfico de esclavos). La cifra manejada para el número total de personas infectadas a nivel mundial ha sido de unos 18 millones según estimaciones previas [7], con el 99% de los casos en África. Más recientemente, y mediante la utilización del mapeo epidemiológico rápido para la oncocercosis (conocido como REMO), se ha logrado obtener estimaciones más precisas de la verdadera extensión de la enfermedad. Este método consiste en seleccionar en cada cuenca fluvial un número de aldeas de acuerdo a métodos apropiados, y cuantificar, en cada comunidad, la prevalencia de nódulos palpables en muestras de personas adultas, a fin de establecer los niveles de endemicidad de la infección en la región [8]. Para finales del 2005, más de 22.000 aldeas habían sido encuestadas con éste método en África (fuera del área cubierta por OCP), permitiendo la identificación de numerosas áreas endémicas desconocidas hasta el momento (Figura 2). Las nuevas poblaciones infectadas así identificadas, junto con su crecimiento demográfico, ciertamente compensan el número de casos prevenidos por OCP (donde se había estimado que unos 3 millones de personas padecían de oncocercosis [7]). Las cifras revisadas consideran que más probablemente, unos 37 millones de individuos son portadores de *O. volvulus* a nivel mundial, y que 90 millones están a riesgo de contraer la infección en África [9].

**Manifestaciones Clínicas y Patogénesis**

La oncocercosis es mejor conocida como “ceguera de los ríos” debido a la elevada prevalencia de ceguera en asentamientos situados a lo largo de ríos de corriente rápida, los cuales proporcionan criaderos favorables para los vectores simúlidos. Hasta unos 500.000 casos de pérdida de la agudeza visual (incluyendo reducción del campo visual), y unos 270.000 casos de ceguera han sido atribuidos a la oncocercosis [7], pero una vez más, estas cifras probablemente constituyen una subestimación de la verdadera magnitud del problema. Las lesiones oculares características de la oncocercosis pueden comprometer todos los tejidos del ojo, desde la cámara anterior (donde se manifiestan en forma de queratitis punteada y esclerosante), hasta la cámara posterior (donde puede producirse atrofia del nervio óptico). Recientemente se ha demostrado, en cohortes de individuos a los cuales se les hizo seguimiento longitudinal durante la implementación de OCP, que la tasa de incidencia de ceguera está asociada con la carga microfilariana que estos individuos habían experimentado en el pasado [10], confirmando el empeoramiento progresivo de la patología ocular que sigue a la exposición crónica con el parásito (Figura 3A). Las lesiones de cámara anterior han sido tradicionalmente atribuidas a una cascada de reacciones inflamatorias provocada por antígenos filarianos [11]. Sin embargo, investigaciones recientes han propuesto que los procesos pro-inflamatorios que conducen a la opacificación de la córnea no son estimulados únicamente por el parásito, sino también por sus bacterias endosimbióticas (pertenecientes al género *Wolbachia*), cuya existencia ha sido demostrada en los últimos años, y las cuales son liberadas en los tejidos por las microfilarias moribundas [12,13]. Esto contrasta con la patogénesis de las lesiones de retina, las cuales pueden proseguir aún después de la eliminación del parásito por el tratamiento, y que pueden resultar de procesos auto-inmunes estimulados por la reactividad cruzada entre el antígeno Ov 39 de *O. volvulus* y el antígeno retiniano humano hr44 [14].

La oncocercosis provoca asimismo marcado prurito y diversas manifestaciones cutáneas que incluyen desde lesiones reactivas tempranas—oncodermatitis papular aguda, oncodermatitis papular crónica, y oncodermatitis liquenificada—hasta lesiones tardías tales como la depigmentación de la piel y la atrofia cutánea [15]. El término ‘sowda’ (‘aswad’ o negro en árabe) se refiere a la condición en la cual la oncodermatitis liquenificada se limita principalmente a una extremidad (oncocercosis localizada). A pesar de las elevadas cargas microfilarianas en piel que generalmente se observan en áreas endémicas, la mayoría de los infectados presentan una dermatitis subclínica o intermitente, que refleja poco ataque celular contra microfilarias vivas (oncocercosis generalizada). En estos casos, las lesiones clínicas corresponden a infiltrados alrededor de microfilarias muertas o en proceso de degeneración que se hallan rodeadas por macrófagos, eosinófilos y neutrófilos [16]. Tal como sucede en la córnea, la inflamación parece ser inducida en gran medida por antígenos derivados de las bacterias endosimbióticas (*Wolbachia*) del parásito [13]. En la oncocercosis generalizada, las reacciones efectoras dependientes de linfocitos T ayudadores (Th) de tipo 1 y 2 (Th1 y Th2), son a su vez suprimidas por un tercer brazo de esta respuesta, específicamente por los linfocitos Th de tipo 3 (también conocidos como linfocitos T reguladores de tipo 1, o Tr-1) [17]. Las células Tr-1 antígeno-específicas constituyen una fuente importante de inter-leuquina 10, lo cual conduce a una supresión del sistema inmunológico que minimiza el daño tisular y maximiza la sobrevivencia del parásito [13]. Al contrario, las personas que padecen de lesiones cutáneas severas o hiperreactivas (oncodermatitis liquenificada o sowda) presentan cargas microfilarianas bajas. En estos casos, las lesiones se producen como resultado de ciclos repetidos de inflamación, infiltración por eosinófilos y macrófagos, y destrucción de microfilarias vivas y muertas [18]. Estos diferentes tipos de respuesta inmunológica podrían estar influenciados por factores genéticos propios del hospedero [19].

La oncocercosis es también una enfermedad sistémica asociada con dolor músculo-esquelético, disminución del índice de masa corporal, y reducción de la productividad laboral. Esto puede deberse a que las microfilarias pueden invadir numerosos tejidos y órganos, y pueden encontrarse en sangre y en orina [5]. También se sospecha que infecciones severas, caracterizadas por elevadas cargas microfilarianas, puedan estar asociadas con el comienzo de ciertos tipos de epilepsia [20], y con el síndrome de enanismo conocido como de Nakalanga [21]. Recientemente, se ha demostrado una asociación directa entre la intensidad de la infección por microfilarias y la mortalidad humana en exceso de la basal en cohortes poblacionales seguidas por OCP [22] (Figura 3B).

**Patrones Epidemiológicos**

A diferencia de las geohelmintiasis y la esquistosomiasis, donde la carga parasitaria alcanza un máximo en los niños y jóvenes en la mayoría de las áreas endémicas, los patrones de edad en la oncocercosis humana muestran una marcada variación que depende de la localidad (la intensidad de la infección por microfilarias de *O. volvulus* puede aumentar, disminuir, o estabilizarse con la edad), y del sexo de los hospedadores. Posibles explicaciones para estos patrones (los cuales tienen implicaciones para la biología poblacional de *O. volvulus* y el diseño de estrategias para su control), comprenden, entre otros, una exposición del hospedero especifica de edad y sexo, la posible operación de factores endocrinos, y la inmunosupresión inducida por el parásito [23,24].

Las razones detrás del establecimiento de OCP en áreas de sabana de 11 países del África occidental estuvieron basadas en la observación de que las poblaciones del parásito en dichas áreas, transmitidas por miembros sabanícolas del complejo *S. damnosum s.l.*, estaban más frecuentemente asociadas con elevadas prevalencias de ceguera que poblaciones del parásito en regiones de bosque, transmitidas por miembros selváticos del vector. Infecciones experimentales cruzadas habían indicado una fuerte adaptación local y un alto grado de incompatibilidad en combinaciones heterólogas, sugiriendo la existencia de complejos *O. volvulus–S. damnosum* que podrían ser responsables por la distribución y severidad diferenciales de la ceguera por oncocercosis [25]. La aplicación subsiguiente de métodos de biología molecular basados en estudios del ADN confirmaron una asociación entre los tipos de parásitos de sabana y selva, con oncocercosis ocular severa o leve, respectivamente [26]. En África occidental, la prevalencia de ceguera en zonas de sabana muestra una correlación positiva y significativa con la intensidad de la infección en la comunidad, un fenómeno que raramente se observa en las áreas de selva [27]. Ello no obstante, la distribución geográfica de lesiones oculares severas y leves, no sigue estrictamente la división entre sabana y selva. Existen áreas de selva, y de mosaico selva-sabana con una elevada prevalencia de ceguera [28] y con tipos del parásito que son distintos de aquellos que se encuentran en África occidental, mientras que en otras regiones, la presencia de parásitos genéticamente indistinguibles de los aislados de sabana no muestran asociación con ceguera [29,30]. Estas diferencias en patogenicidad podrían deberse a diferencias en las concentraciones relativas de *Wolbachia* [31] en las diversas cepas del parásito.

**La Carga de Enfermedad y Consecuencias Socioeconómicas**

La verdadera magnitud de la carga de enfermedad por oncocercosis ha sido indudablemente subestimada. La mortalidad en exceso de los ciegos, y particularmente entre los hombres, puede ser considerable [32,33]. Aún en aquellos individuos videntes, una carga microfilariana elevada puede reducir la expectativa de vida [22]. La inmunosupresión frente antígenos tanto específicos de *O. volvulus* como no-específicos [34], la disminución de la capacidad de luchar contra infecciones y de seroconvertir exitosamente luego de vacunaciones [35], y la asociación entre infección severa y epilepsia [20], pueden ser parcialmente responsables por la mortalidad en exceso. Asimismo, es bien conocido que la oncodermatitis y la epilepsia están asociadas con una estigmatización social importante [36]. Se considera que la oncocercosis es responsable por la pérdida de aproximadamente 1 millón de años de vida ajustados por incapacitación (DALY)—años de vida saludable perdidos por incapacitación y mortalidad (con más de la mitad de los mismos debido a lesiones cutáneas [37]). Esta carga de enfermedad disminuye en gran medida la capacidad de generar ingresos [38], repercute en costos importantes para los servicios sanitarios, y ejerce un impacto socioeconómico negativo sobre las poblaciones afectadas y el uso de la tierra [39]. Si bien la oncocercosis no es la única causa del despoblamiento de valles fértiles en África occidental, ciertamente ha dificultado la recolonización de tierras arables [40]. Los beneficios acumulados a través de los programas de control de la oncocercosis deberían medirse no sólo en relación con el número de casos de ceguera prevenidos y la relación costo-efectividad del tratamiento administrado [41,42], sino también en términos del número de muertes evitadas.

**Estrategias de Control de la Oncocercosis**

La lucha contra la oncocercosis de basa esencialmente en estrategias para la eliminación del vector (medidas antivectoriales), y la utilización de medicamentos contra el parásito (medidas antiparasitarias). Las primeras están dirigidas contra los estadios acuáticos de los simúlidos vectores, y las últimas en contra de las microfilarias. Hasta el momento, no existe un fármaco macrofilaricida eficaz y seguro para su administración masiva. El programa de OCP comenzó por implementar rociamientos semanales de los criaderos del vector con larvicidas a fin de interrumpir la transmisión en el área central del programa. Una vez logrado esto, la eliminación del parásito requiere de la abolición de las fuentes de nuevos vectores mientras se encuentren microfilarias en piel. Dicha duración fue estimada en un mínimo de 14 años (considerando la expectativa de vida de los vermes adultos y de las microfilarias) [43]. En algunas áreas de OCP, pudo demostrarse que aquellos niños nacidos después del comienzo del control del vector, no desarrollaron la infección [44]. En 1987, Merck tomó la decisión sin precedentes de donar la ivermectina (Mectizan), un medicamento microfilaricida efectivo y seguro para el tratamiento masivo, por cuanto tiempo fuere necesario para eliminar la oncocercosis como problema de salud pública. A partir de ese momento se introdujo, en algunas áreas de OCP, la distribución regular del tratamiento (administrado por equipos móviles), como medida complementaria de la lucha antivectorial, y como única medida de control en otras áreas del programa [45]. La ivermectina, administrada en dosis de 150 microgramos por kilogramo de peso, posee una marcada actividad microfilaricida e inhibe la liberación de microfilarias por parte de los gusanos hembra adultos por varios meses. La administración masiva de ivermectina una o dos veces por año (a todos aquellos por encima de los cinco años de edad, excluyendo a los niños que pesen menos de 15 kg, y las mujeres embarazadas o amamantando a un recién nacido de menos e una semana), reduce la morbilidad y previene la incapacitación [46,47] además de disminuir la transmisión [48,49]. Ya que la endemicidad inicial de algunos focos es sumamente elevada, se considera que los regímenes de distribución anual no serían suficientes para lograr la eliminación local de poblaciones del parásito [50], a menos que se alcancen y mantengan niveles de cobertura del tratamiento por encima del 80% de la población total durante un mínimo de 25 años, y contando con que no se produzca una disminución de la eficacia del tratamiento [51].

En Latinoamérica, y particularmente en Guatemala actividades más limitadas y focales de control de vectores fueron conducidas con cierto grado de éxito en contra del vector *S. ochraceum* s.l. [52], pero en general la lucha antivectorial se considera impráctica en este continente. El Programa para la Eliminación de la Oncocercosis en las Américas (OEPA) fue iniciado en 1993 como una iniciativa regional para eliminar la morbilidad por oncocercosis (y suprimir la transmisión donde sea posible) en los focos de los seis países endémicos [53]. La estrategia de OEPA se basa actualmente en la distribución bianual de ivermectina, por considerarse que el tratamiento masivo cada seis meses tendría un mayor impacto sobre la transmisión [54] y la fecundidad de las hembras adultas [55].

En 1995, el Programa Africano para el Control de la Oncocercosis (APOC) fue establecido a fin de cubrir los restantes 19 países africanos que no habían formado parte de OCP [56]. (Tres de estos países, Kenya, Rwanda y Mozambique resultaron no ser endémicos.) Desde su inauguración , la estrategia de APOC se ha basado en la distribución de ivermectina una vez por año. Los niveles de cobertura geográfica (porcentaje de comunidades tratadas en un área) y terapéutica (porcentaje de la población tratada en una comunidad) que se alcanzaron inicialmente a través de los equipos móviles resultaron ser insatisfactorios y demostraron poca sostenibilidad. En su lugar, esta estrategia de distribución fue reemplazada, con gran éxito, por la modalidad de tratamiento con ivermectina dirigido por las comunidades (TIDC), mediante la cual las mismas comunidades designan a sus distribuidores locales responsables [57]. Para fines del 2005, 400 millones de tratamientos habían sido suministrados por el Programa de Donación de Mectizan, y cerca de 40 millones de personas, viviendo en 90.000 aldeas africanas, fueron tratadas por aproximadamente 300.000 distribuidores comunitarios a lo largo de los proyectos patrocinados por APOC.

El costo promedio por persona tratada, incluyendo el tiempo de los voluntarios, es de 0,74 centavos de dólar, por lo que el TIDC resulta altamente costo-efectivo [9]. Además, el costo por persona tratada como parte de APOC (excluyendo el precio del Mectizan) es casi 8,5 veces más económico que el costo por persona protegida a través del control vectorial por parte de OCP [42]. Por otro lado, la estrategia de TIDC ha devuelto el poder de decisión a las comunidades hasta tal grado, que actualmente está siendo utilizada como plataforma para integrar otras intervenciones comunitarias tales como suplementación con vitamina A, y tratamiento de filariasis linfática con albendazol. Esta integración con otros programas de control podría ayudar a mantener los altos niveles de cobertura que se requieren a largo plazo, una vez que los síntomas de la oncocercosis mejoran [58]. Sin embargo, y a pesar de tales e impresionantes logros en términos de cobertura, y de las perspectivas promisorias de intervenciones combinadas dirigidas por la comunidad, APOC debe confrontar serios retos a fin de lograr sus metas últimas de tratamiento, de sostenibilidad a largo plazo, y de impacto substancial permanente.

En aquellas regiones donde la oncocercosis y la loiasis (causada por la filaria *Loa loa*) son coendémicas (principalmente en África central), el tratamiento de la oncocercosis por ivermectina puede resultar en eventos adversos severos, incluyendo encefalopatía fatal [59]. Esto ha representado un obstáculo importante para la expansión de APOC, y ha motivado tanto el desarrollo de modelos geoestadísticos que tienen como objeto identificar áreas en África donde existe un alto riesgo de que la loiasis sea severa [60], como el diseño de protocolos de tratamiento dirigidos a reducir la microfilaremia por *L loa* previamente al tratamiento con ivermectina.

Los estudios que han evaluado la sostenibilidad de los proyectos patrocinados por APOC han revelado que no siempre las comunidades apoyan a sus distribuidores de una manera adecuada, siendo otras actividades más “lucrativas”, tal como la participación de los mismos en las campanas de inmunización, lo que mantiene su compromiso en lugar de TIDC per se. La falta de recursos dificulta en muchos casos las actividades de supervisión en los niveles comunitarios y de estructuras de salud, lo cual supone obstáculos importantes que deben ser superados a fin de integrar TIDC exitosamente con otras actividades sanitarias [61].

De todos los aspectos anteriormente mencionados surge la pregunta crucial de por cuánto tiempo debe durar APOC. En sus inicios, se pensó que la duración de APOC sería de 12 años (desde 1995 hasta el 2007). Desde entonces, se ha añadido una fase de devolución de 2 años, y se ha asegurado el apoyo de los donantes hasta el 2010. Hasta ahora, no se han tomado decisiones sobre la posibilidad de otras extensiones de su duración, pero dados los ciclos de vida del parásito y vector, se necesitaría que las actividades de APOC fueran sostenidas al menos por 20 años a fin de ejercer un efecto significativo y perdurable [42].

**Necesidad de Otros Medicamentos Eficaces en contra de *O. volvulus***

La dependencia creciente del control de la oncocercosis sobre la ivermectina como fármaco único para su tratamiento, y la ausencia de un avance significativo en el desarrollo de vacunas efectivas [62], han incentivado la investigación sobre otros compuestos con actividad antiparasitaria. Entre estos, la moxidectina actúa como un fármaco microfilaricida eficaz, cuyo promedio de vida en el cuerpo humano es más largo que el de la ivermectina [63], y es posible, por consiguiente, que pueda suprimir la fecundidad de las hembras adultas por un período más prolongado [63]. La estructura química de la moxidectina es similar a la de la ivermectina, y en modelos animales no parece tener acción macrofilaricida [64].

Ya que la eliminación prolongada de *Wolbachia* perjudica la reproducción y sobrevivencia de los vermes adultos [65], se ha sugerido que nuevas intervenciones quimioterapéuticas podrían basarse en el uso de antibióticos en contra de estas bacterias. Regímenes de tratamiento diarios con 100 miligramos de doxiciclina por seis semanas (o 200 miligramos diarios por cuatro semanas), producen una interrupción de la embriogénesis de *O. volvulus* por un mínimo de 18 meses [66]. Sin embargo, la prolongada duración del tratamiento, las variadas contraindicaciones de los antibióticos, y el riesgo de inducir resistencia en otros patógenos, dificultan la incorporación de estos regímenes en los actuales programas de tratamiento masivo. A fin de superar estos obstáculos, se están conduciendo estudios a fin de investigar la eficacia de otros antibióticos, e identificar los cursos de tratamiento más cortos que puedan remover efectiva y permanentemente las bacterias [67]. Alternativamente, la terapia anti-*Wolbachia* podría ser utilizada para tratar selectivamente aquellos individuos identificados como positivos por microfilarias hacia el final de programas de distribución masiva de ivermectina, con el objetivo de “limpiar” áreas donde se considere que la eliminación del parásito es posible.

Es de esperarse que con la expansión de todos los programas que dependen de la ivermectina (los países que previamente pertenecían a OCP, y aquellos en APOC y OEPA), se impongan presiones selectivas sobre el genoma del parásito. Si bien aún no se han confirmado casos de resistencia a la ivermectina, se ha reportado la existencia de un fenotipo de respuesta subóptima al tratamiento en individuos residentes en algunas localidades de Ghana que han experimentado más de nueve rondas de tratamiento [68]. La explicación de este fenómeno no parece radicar en una pérdida de la eficacia microfilaricida, sino en una recuperación temprana de la actividad reproductiva por parte de las hembras adultas. Además de estas observaciones, se han realizado análisis genéticos de vermes obtenidos de individuos que habían recibido seis o más dosis anuales de ivermectina en comparación con aquellos que no habían recibido tratamiento. Los resultados indican que en áreas con presión quimioterapéutica por ivermectina, se está produciendo selección sobre genes polimórficos del genoma de *O. volvulus* (los cuales, en nemátodos de importancia veterinaria están asociados con la resistencia a la ivermectina) [69]. Ello no obstante, aún no se han conducido los estudios definitivos que investiguen la relación entre cambios fenotípicos y genotípicos en poblaciones parasitarias en función de un creciente número de dosis de tratamiento. Los modelos matemáticos pueden contribuir a comprender aspectos de la biología poblacional del parásito que determinan las tasas de recrudescencia de la infección [70,71] y la propagación de alelos favorablemente seleccionados por presión quimioterapéutica con ivermectina.

**Modelos Matemáticos para el Control de la Oncocercosis**

La oncocercosis es uno de los mejores ejemplos en la historia del control de las enfermedades parasitarias donde los modelos de simulación han proporcionado información importante en todas las etapas de la intervención. ONCHOSIM, en particular, fue desarrollado bajo los auspicios de OCP para el modelaje de la oncocercosis en zonas de sabana de África occidental [72]. Otros modelos han sido construidos para describir la dinámica de transmisión en áreas de selva, y en focos de Latinoamérica [73]. La pregunta clave de por cuánto tiempo debe administrarse el tratamiento antifilariano depende de las metas trazadas por los programas y de la características epidemiológicas específicas de cada foco. Si el objetivo es el de eliminar la oncocercosis como problema de salud pública, la administración anual de ivermectina en los países de APOC representará una estrategia exitosa una vez que los niveles de infección en las comunidades se reduzcan por debajo de cinco a diez microfilarias por biopsia de piel. Esto, sin embargo, no logrará interrumpir la transmisión de *O. volvulus* en África [74]. Factores tales como la intensidad y la estacionalidad de la transmisión, los complejos *Onchocerca–Simulium* presentes, la distribución del parásito en la población de hospedadores, los procesos denso-dependientes que operan en el ciclo de vida del parásito, y la interacción de todo lo anterior con la intervención y su cobertura, determinará la estabilidad del sistema hospedador*–*parásito y nuestra capacidad (o incapacidad) de conducir a las poblaciones de *O. volvulus* por debajo de posibles puntos de ruptura de la transmisión [70,71,73].

**Conclusión**

El desinterés por la lucha contra la oncocercosis puede manifestarse de muchas maneras. Se requiere de un compromiso económico y político no solamente para apoyar los programas de control, sino también para financiar la necesaria investigación que pueda proveer los instrumentos que posibiliten la eliminación del parásito. El éxito espectacular de OCP ha empujado a la oncocercosis hacia el fondo de las agendas de investigación en salud en un momento crucial, cuando consolidar sus logros, proteger su inversión, y demostrar el éxito a largo plazo de APOC y OEPA son de la mayor importancia. Se deberá dar prioridad al desarrollo de herramientas para mejorar el diagnóstico (la detección de microfilarias en biopsias de piel perderá sensibilidad a medida que los programas de control avanzan, y el desarrollo de pruebas para la detección de antígeno parasitario ha demostrado ser dificultoso); la búsqueda de compuestos macrofilaricidas; la detección temprana de la potencial pérdida de eficacia de la ivermectina y de cambios en la estructura genética del parásito, y una mejor comprensión del impacto del tratamiento sobre la biología poblacional de *O. volvulus*. Las perspectivas actuales de distribución de ivermectina por un período indefinido conlleva el riesgo de aparición de resistencia y el cansancio de las poblaciones afectadas y los donantes.

**Agradecimientos**

Las ideas discutidas en el presente artículo se han beneficiado del apoyo financiero, a lo largo de los años por parte del Wellcome Trust (MGB), el Medical Research Council del Reino Unido (MGB y TSC), la Fondation pour la Recherche Médicale y la Fondation Singer-Polignac, Francia (SDSP), la Fundación para la Ceguera de los Ríos, y el Programa Especial de la UNICEF/PNUD/Banco Mundial/OMS para la Investigación y Entrenamiento en Enfermedades Tropicales, TDR (MB).

**Bibliografía**

1. <http://www.who.int/dg/speeches/2002/Ouagadougou/en>

2. Hopkins AD (2005) Ivermectin and onchocerciasis: Is it all solved? Eye 19: 1057–1066.

3. Crosskey RW (1990) The natural history of blackflies. Chichester: John Wiley & Sons. 711 p.

4. Crosskey RW, Howard TM (2004) A revised taxonomic and geographical inventory of world blackflies (Diptera : Simuliidae). London: The Natural History Museum. Available: http://www.nhm.ac.uk/research-curation/projects/blackflies. Accessed 24 April 2006.

5. Bradley JE, Whitworth J, Basáñez M-G (2005) Onchocerciasis. In: Cox FEG, Wakelin D, Gillespie SH, Despommier DD. Parasitology, Topley and Wilson’s microbiology and microbial infections. 10th edition. London: Hodder Arnold. pp. 781–801.

6. World Health Organization (2005) Onchocerciasis (river blindness). Weekly epidemiological record 80: 257–260. Available: http://www.who.int/wer/2005/wer8030.pdf. Accessed 26 July 2006.

7. World Health Organization (1995) Onchocerciasis and its control. Report of a WHO expert committee on onchocerciasis control. WHO Technical Report Series, number 852. Geneva: World Health Organization. 110 p.

8. Ngoumou P, Walsh JF, Macé JM (1994) A rapid mapping technique for the prevalence and distribution of onchocerciasis: A Cameroon case study. Ann Trop Med Parasitol 88: 463–474.

9. African Programme for Onchocerciasis Control [APOC] (2005) Final communiqué of the 11th session of the Joint Action Forum (JAF) of APOC, Paris, France, 6–9 December 2005. Ouagadougou (Burkina Faso): APOC.

10. Little MP, Basáñez M-G, Breitling LP, Boatin BA, Alley ES (2004) Incidence of blindness during the entire duration of the Onchocerciasis Control Programme in western Africa, 1971–2002. J Infect Dis 189: 1932–1941.

11. Hall LR, Pearlman E (1999) Pathogenesis of onchocercal keratitis (river blindness). Clin Microbiol Rev 12: 445–453.

12. Saint-André A, Blackwell NM, Hall LR, Hoerauf A, Brattig NW, et al. (2002) The role of endosymbiotic *Wolbachia* bacteria in the pathogenesis of river blindness. Science 295: 1892–1895.

13. Brattig NW (2004) Pathogenesis and host responses in human onchocerciasis: Impact of *Onchocerca* filariae and *Wolbachia* endobacteria. Microbes Infect 6: 113–128.

14. McKechnie NM, Gürr W, Yamada H, Copland D, Braun G (2002) Antigenic mimicry: *Onchocerca volvulus* antigen-specific T cells and ocular inflammation. Invest Ophthalmol Vis Sci 43: 411–418.

15. Murdoch ME, Hay RJ, Mackenzie CD, Williams JF, Ghalib HW, et al. (1993) A clinical classification and grading system of the cutaneous changes in onchocerciasis. Br J Dermatol 129: 260–269.

16. Pearlman E, Garhart CA, Grand DJ, Diaconu E, Strine ER, et al. (1999) Temporal recruitment of neutrophils and eosinophils to the skin in a murine model for onchocercal dermatitis. Am J Trop Med Hyg 61: 14–18.

17. Hoerauf A, Brattig N (2002) Resistance and susceptibility in human onchocerciasis—Beyond Th1 vs. Th2. Trends Parasitol 18: 25–31.

18. Ali MM, Baraka OZ, AbdelRahman SI, Sulaiman SM, Williams JF, et al. (2003) Immune responses directed against microfilariae correlate with severity of clinical onchodermatitis and treatment history. J Infect Dis 187: 714–717.

19. Meyer CG, Gallin M, Erttmann KD, Brattig N, Schnittger L, et al. (1994) HLA-D alleles associated with generalized disease, localized disease, and putative immunity in *Onchocerca volvulus* infection. Proc Natl Acad Sci U S A 91: 7515–7519.

20. Boussinesq M, Pion SD, Demanga-Ngangue, Kamgno J (2002) Relationship between onchocerciasis and epilepsy: A matched case-control study in the Mbam Valley, Republic of Cameroon. Trans R Soc Trop Med Hyg 96: 537–541.

21. Kipp W, Burnham G, Bamuhiiga J, Leichsenring M (1996) The Nakalanga syndrome in Kabarole district, Western Uganda. Am J Trop Med Hyg 54: 80–83.

22. Little MP, Breitling LP, Basáñez M-G, Alley ES, Boatin BA (2004) Association between microfilarial load and excess mortality in human onchocerciasis: An epidemiological study. Lancet 363: 1514–1521.

23. Filipe JA, Boussinesq M, Renz A, Collins RC, Vivas-Martinez S, et al. (2005) Human infection patterns and heterogeneous exposure in river blindness. Proc Natl Acad Sci U S A 102: 15265–15270.

24. Duerr HP, Dietz K, Schulz-Key H, Büttner DW, Eichner M (2003) Density-dependent parasite establishment suggests infection-associated immunosuppression as an important mechanism for parasite density regulation in onchocerciasis. Trans R Soc Trop Med Hyg 97: 242−250.

25. Duke BO, Lewis DJ, Moore PJ (1966) *Onchocerca-Simulium* complexes. I. Transmission of forest and Sudan-savanna strains of *Onchocerca volvulus*, from Cameroon, by *Simulium damnosum* from various West African bioclimatic zones. Ann Trop Med Parasitol 60: 318–336.

26. Zimmerman PA, Dadzie KY, De Sole G, Remme J, Alley ES, et al. (1992) *Onchocerca volvulus* DNA probe classification correlates with epidemiologic patterns of blindness. J Infect Dis 165: 964–968.

27. Dadzie KY, Remme J, Rolland A, Thylefors B (1989) Ocular onchocerciasis and intensity of infection in the community. II. West African rainforest foci of the vector *Simulium yahense*. Trop Med Parasitol 40: 348–354.

28. Kayembe DL, Kasonga D L, Kayembe PK, Mwanza JC, Boussinesq M (2003) Profile of eye lesions and vision loss: A cross-sectional study in Lusambo, a forest-savanna area hyperendemic for onchocerciasis in the Democratic Republic of Congo. Trop Med Int Health 8: 83–89.

29. Fischer P, Bamuhiiga J, Kilian AH, Büttner DW (1996) Strain differentiation of *Onchocerca volvulus* from Uganda using DNA probes. Parasitology 112: 401–408.

30. Higazi TB, Katholi CR, Mahmoud BM, Baraka OZ, Mukhtar MM, et al. (2001) *Onchocerca volvulus*: Genetic diversity of parasite isolates from Sudan. Exp Parasitol 97: 24–34.

31. Higazi TB, Filiano A, Katholi CR, Dadzie Y, Remme JH, et al. (2005) *Wolbachia* endosymbiont levels in severe and mild strains of *Onchocerca volvulus*. Mol Biochem Parasitol 141: 109–112.

32. Prost A (1986) The burden of blindness in adult males in the savanna villages of West Africa exposed to onchocerciasis. Trans R Soc Trop Med Hyg 80: 525–527.

33. Pion SD, Kamgno J, Demanga-Ngangue, Boussinesq M (2002) Excess mortality associated with blindness in the onchocerciasis focus of the Mbam Valley, Cameroon. Ann Trop Med Parasitol 96: 181–189.

34. Stewart GR, Boussinesq M, Coulson T, Elson L, Nutman T, et al. (1999) Onchocerciasis modulates the immune response to mycobacterial antigens. Clin Exp Immunol 117: 517–523.

35. Cooper PJ, Espinel I, Paredes W, Guderian RH, Nutman TB (1998) Impaired tetanus-specific cellular and humoral responses following tetanus vaccination in human onchocerciasis: A probable role for interleukin-10. J Infect Dis 178: 1133–1138.

36. Vlassoff C, Weiss M, Ovuga EB, Eneanya C, Newel PT, et al. (2000) Gender and the stigma of onchocercal skin disease in Africa. Soc Sci Med 50: 1353–1368.

37. Remme JH (2004) Research for control: The onchocerciasis experience. Trop Med Int Health 9: 243–254.

38. Oladepo O, Brieger WR, Otusanya S, Kale OO, Offiong S, et al. (1997) Farm land size and onchocerciasis status of peasant farmers in south-western Nigeria. Trop Med Int Health 2: 334–340.

39. Evans TG (1995) Socioeconomic consequences of blinding onchocerciasis. Bull World Health Organ 73: 495–506.

40. Hervouët JP, Prost A (1979) Organisation de l'espace et épidémiologie de l'onchocercose. In: Maîtrise de l'espace agraire et développement en Afrique tropicale. Mémoires ORSTOM, number 89. Paris: ORSTOM. pp. 179–190.

41. Benton B (1998) Economic impact of onchocerciasis control through the African Programme for Onchocerciasis Control: An overview. Ann Trop Med Parasitol 92 (Suppl): S33–S39.

42. Waters HR, Rehwinkel JA, Burnham G (2004) Economic evaluation of Mectizan distribution. Trop Med Int Health 9 (Suppl): A16–A25.

43. Hougard JM, Alley ES, Yaméogo L, Dadzie KY, Boatin BA (2001) Eliminating onchocerciasis after 14 years of vector control: A proved strategy. J Infect Dis 184: 497−503.

44. Ba O, Karam M, Remme J, Zerbo G (1987) Place des enfants dans l'évaluation du programme de lutte contre l'onchocercose en Afrique de l'ouest [Role of children in the evaluation of the Onchocerciasis Control Program in West Africa]. Trop Med Parasitol 38: 137–142.

45. Molyneux DH (1995) Onchocerciasis control in West Africa: Current status and future of the Onchocerciasis Control Programme. Parasitol Today 11: 399–402.

46. Ejere H, Schwartz E, Wormald R (2001) Ivermectin for onchocercal eye disease (river blindness). Cochrane Database Syst Review: 2001: CD002219.

47. Tielsch JM, Beeche A (2004) Impact of ivermectin on illness and disability associated with onchocerciasis. Trop Med Int Health 9 (Suppl): A45–A56.

48. Boussinesq M, Prod'hon J, Chippaux JP (1997) *Onchocerca volvulus*: Striking decrease in transmission in the Vina valley (Cameroon) after eight annual large scale ivermectin treatments. Trans R Soc Trop Med Hyg 91: 82−86.

49. Collins RC, Gonzalez-Peralta C, Castro J, Zea-Flores G, Cupp MS, et al. (1992) Ivermectin: Reduction in prevalence and infection intensity of *Onchocerca volvulus* following biannual treatments in five Guatemalan communities. Am J Trop Med Hyg 47: 156−169.

50. Borsboom GJ, Boatin BA, Nagelkerke NJ, Agoua H, Akpoboua KL, et al. (2003) Impact of ivermectin on onchocerciasis transmission: Assessing the empirical evidence that repeated ivermectin mass treatments may lead to elimination/eradication in West Africa. Filaria J 2: 8.

51. Winnen M, Plaisier AP, Alley ES, Nagelkerke NJ, van Oortmarssen G, et al. (2002) Can ivermectin mass treatments eliminate onchocerciasis in Africa? Bull World Health Organ 80: 384–390.

52. Ochoa JO, Castro JC, Barrios VM, Juarez EL, Tada I (1997) Successful control of onchocerciasis vectors in San Vicente Pacaya, Guatemala, 1984–1989. Ann Trop Med Parasitol 91: 471−479.

53. Richards FO, Boatin B, Sauerbrey M, Sékétéli A (2004) Control of onchocerciasis today: Status and challenges. Trends Parasitol 17: 558–563.

54. Cupp EW, Ochoa JO, Collins RC, Cupp MS, Gonzalez-Peralta C, et al. (1992) The effects of repetitive community-wide ivermectin treatment on transmission of *Onchocerca volvulus* in Guatemala. Am J Trop Med Hyg 47: 170−180.

55. Duke BO, Zea-Flores G, Castro J, Cupp EW, Muñoz B (1991) Comparison of the effects of a single dose and of four six monthly doses of ivermectin on adult *Onchocerca volvulus*. Am J Trop Med Hyg 45: 132−137.

56. Remme JHF (1995) The African Programme for Onchocerciasis Control: Preparing to launch. Parasitol Today 11: 403–406.

57. Amazigo UV, Obono OM, Dadzie KY, Remme J, Jiya J, et al. (2002) Monitoring community-directed treatment programmes for sustainability: Lessons from the African Programme for Onchocerciasis Control (APOC). Ann Trop Med Parasitol 96 (Suppl 1): S75−S92.

58. Molyneux DH (2005) Onchocerciasis control and elimination: Coming of age in resource-constrained health systems. Trends Parasitol 21: 525−529.

59. Gardon J, Gardon-Wendel N, Demanga-Ngangue, Kamgno J, Chippaux JP, et al. (1997) Serious reactions after mass treatment of onchocerciasis with ivermectin in an area endemic for *Loa loa* infection. Lancet 350: 18–22.

60. Thomson MC, Obsomer V, Kamgno J, Gardon J, Wanji S, et al. (2004) Mapping the distribution of *Loa loa* in Cameroon in support of the African Programme for Onchocerciasis Control. Filaria J 3: 7.

61. African Programme for Onchocerciasis Control [APOC] (2005) External evaluation report. Ouagadougou (Burkina Faso): APOC.

62. Cook JA, Steel C, Ottesen EA (2001) Towards a vaccine for onchocerciasis. Trends Parasitol 17: 555−558.

63. Cotreau MM, Warren S, Ryan JL, Fleckenstein L, Vanapalli SR, et al. (2003) The antiparasitic moxidectin: Safety, tolerability, and pharmacokinetics in humans. J Clin Pharmacol 43: 1108–1115.

64. Trees AJ, Graham SP, Renz A, Bianco AE, Tanya V (2000) *Onchocerca ochengi* infections in cattle as a model for human onchocerciasis: Recent developments. Parasitology 120 (Suppl): S133–S142.

65. Gilbert J, Nfon CK, Makepeace BL, Njongmeta LM, Hastings IM, et al. (2005) Antibiotic chemotherapy of onchocerciasis: In a bovine model, killing of adult parasites requires a sustained depletion of endosymbiotic bacteria (*Wolbachia* species). J Infect Dis 192: 1483–1493.

66. Hoerauf A, Büttner DW, Adjei O, Pearlman E (2003) Onchocerciasis. BMJ 326: 207–210.

67. Taylor MJ, Hoerauf A (2001) A new approach to the treatment of filariasis. Curr Opin Infect Dis 14: 727–731.

68. Awadzi K, Boakye DA, Edwards G, Opoku NO, Attah SK, et al. (2004) An investigation of persistent microfilaridermias despite multiple treatments with ivermectin, in two onchocerciasis-endemic foci in Ghana. Ann Trop Med Parasitol 98: 231–249.

69. Eng JK, Prichard RK (2005) A comparison of genetic polymorphism in populations of *Onchocerca volvulus* from untreated- and ivermectin-treated patients. Mol Biochem Parasitol 142: 193–202.

70. Churcher TS, Ferguson NM, Basáñez M-G (2005) Density dependence and overdispersion in the transmission of helminth parasites. Parasitology 131: 121–132.

71. Duerr HP, Dietz K, Eichner M (2005) Determinants of the eradicability of filarial infections: A conceptual approach. Trends Parasitol 21: 88−96.

72. Habbema JD, Alley ES, Plaisier AP, van Oortmarssen GJ, Remme JH (1992) Epidemiological modelling for onchocerciasis control. Parasitol Today 8: 99–103.

73. Basáñez M-G, Ricárdez-Esquinca J (2001) Models for the population biology and control of human onchocerciasis. Trends Parasitol 17: 430–438.

74. Dadzie Y, Neira M, Hopkins D (2003) Final report of the Conference on the Eradicability of Onchocerciasis. Filaria J 2: 2.

75. African Programme for Onchocerciasis Control (2004) Year 2004 Progress Report of WHO/APOC. Available: http://www.apoc.bf/en/download.htm. Accessed 12 January 2006.

76. World Health Organization/African Programme for Onchocerciasis Control (2002) Onchocerciasis control in special intervention zones including Sierra Leone in the OCP area. Joint Programme Committee Report, September 2002. Ouagadougou (Burkina Faso): World Health Organization/African Programme for Onchocerciasis Control.

**Figura 1.** Ciclo de vida de *O. volvulus*.

(Ilustración: Giovanni Maki, derivada de una imagen del CDC. http://www.dpd.cdc.gov/dpdx/HTML/Filariasis.htm)

DOI: 10.1371/journal.pmed.0030371.g001

**Figura 2.** Mapa de la Distribución de la Oncocercosis indicando el Estado Actual de los Programas de Control.

Las regiones en rojo indican aquellas zonas donde actualmente se distribuye tratamiento con ivermectina. Las regiones en amarillo muestran las zonas donde hace falta realizar encuestas epidemiológicas suplementarias. La región en verde representa el área cubierta por el programa de lucha contra la oncocercosis en África occidental (OCP). Las regiones violáceas corresponden a Zonas Especiales de Intervención, correspondientes a zonas anteriormente bajo OCP donde se organizan tratamientos con ivermectina y prosigue cierto grado de control vectorial [53,75,76].

DOI: 10.1371/journal.pmed.0030371.g002

**Figura 3.** Tasa de Incidencia de Ceguera y Tasa de Mortalidad en Exceso por Edad y Sexo, en Función de la Carga Microfilariana de *O. volvulus*.

Media aritmética del número de microfilarias obtenido por biopsia cutánea en crestas ilíacas derecha e izquierda, utilizando pinza esclerocorneal de Holth de 2 mm. (A) Ceguera; (B) Tasa de mortalidad en exceso. Las barras indican los intervalos de confianza del 95% [10,22].

DOI: 10.1371/journal.pmed.0030371.g003
